# Multi‐Functional Adaptive Interfaces for Next‐Generation Wearable and Implantable Bioelectronics

**DOI:** 10.1002/advs.202600043

**Published:** 2026-03-28

**Authors:** Jinhong Park, Junyoung Ha, Do Gyun Kim, Soo‐Hong Lee, Hyun‐Do Jung, Ja Hoon Koo, Gi Doo Cha, Dong Chan Kim

**Affiliations:** ^1^ The Artie McFerrin Department of Chemical Engineering Texas A&M University College Station Texas USA; ^2^ Department of Chemical, Biological, and Battery Engineering Gachon University Gyeonggi‐do Republic of Korea; ^3^ Department of Semiconductor Engineering Gachon University Gyeonggi‐do Republic of Korea; ^4^ Department of Systems Biotechnology Chung‐Ang University Anseong Republic of Korea; ^5^ Department of Medical Biotechnology Dongguk University Gyeonggi Republic of Korea; ^6^ Division of Materials Science and Engineering Hanyang University Seoul Republic of Korea; ^7^ Department of Semiconductor Systems Engineering and Institute of Semiconductor and System IC Sejong University Seoul Republic of Korea

**Keywords:** adaptive interfaces, bioelectronics, human‐machine interfaces, wearable and implantable electronics

## Abstract

Healthcare systems are progressively shifting toward long‐term, personalized models that rely on continuous physiological monitoring, thereby intensifying the need for advanced bioelectronic technologies. The evolution of bioelectronics can be described in three generations. First‐generation systems, constructed from rigid materials, exhibited severe mechanical mismatch that produced interfacial gaps and provoked inflammatory responses when interfaced with soft tissues. Second‐generation platforms based on soft, stretchable materials addressed many of these limitations by improving conformality and reducing mechanical stress; however, maintaining stable, long‐term attachment to dynamically moving tissues remained a major barrier to clinical translation. Consequently, recent research focused on third‐generation bioelectronics that couple intrinsically soft electronic materials with multifunctional, adaptive human–machine interfaces capable of responding to both mechanical and biochemical cues within the body. In this review, we summarize recent advances in such adaptive interfaces, categorized into mechano‐adaptive and biophysiologically adaptive modalities. We first examine mechano‐adaptive strategies, including shape programmability, injectability, anti‐swelling architectures, and self‐healing mechanisms, followed by biophysiologically adaptive approaches such as controlled permeability, anti‐fibrotic design, tissue adhesion, and biodegradability. We then highlight representative wearable and implantable systems that incorporate these adaptive concepts, and conclude by discussing key challenges and future directions required for advancing these technologies toward stable, long‐term clinical use.

## Graphical abstract

Adaptive Biointerfaces: A comprehensive overview of next‐generation bioelectronics is presented, highlighting mechano‐adaptive and biophysiologically adaptive interfaces that enable stable, long‐term integration with living tissues. As illustrated in the image, adaptive interface strategies, including mechano‐adaptivity (e.g., injectability, shape programmability, anti‐swelling, and self‐healing), and biophysiological adaptivity (e.g., permeability, anti‐fibrotic design, adhesion, and biodegradability), provide multifunctional pathways toward next‐generation wearable and implantable bioelectronic systems.

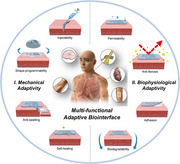

## 1 Introduction

**FIGURE 1:**
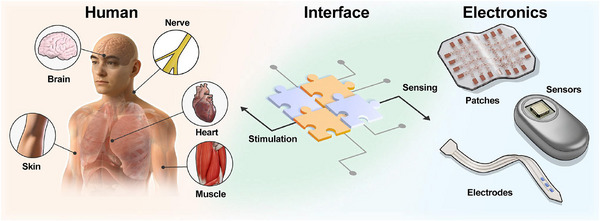
Schematic representation of the features of wearable and implantable bioelectronic technologies, including human, electronics, and their bridging interface.

As the global elderly population grows and chronic diseases become increasingly prevalent, healthcare systems are shifting toward long‐term, patient‐centered models that emphasize continuous and personalized care. This transition is accelerating the movement from conventional hospital‐based treatment to at‐home, self‐managed healthcare supported by real‐time physiological monitoring [1, 2, 3]. Central to this transformation are recent advances in soft bioelectronic technologies (Figure 1). Evolving from bulky, stationary biomedical instruments, modern bioelectronics now offer lightweight, mechanically durable, and biocompatible platforms capable of conformally integrating with diverse tissues (including the brain, heart, nerves, muscles, and skin) to record dynamic physiological signals and deliver targeted stimulations [4, 5, 6, 7]. When coupled with emerging Internet of Things (IoT) infrastructures and artificial intelligence (AI) algorithms, these systems can continuously analyze large physiological datasets, enabling early detection of pathological changes, personalized therapeutic feedback, and proactive disease management [8, 9]. By reducing temporal and spatial barriers inherent to conventional clinical care, such technologies hold substantial promise for improving accessibility, lowering healthcare burden, and enhancing quality of life through timely interventions and autonomous medical support [10, 11].

**FIGURE 2:**
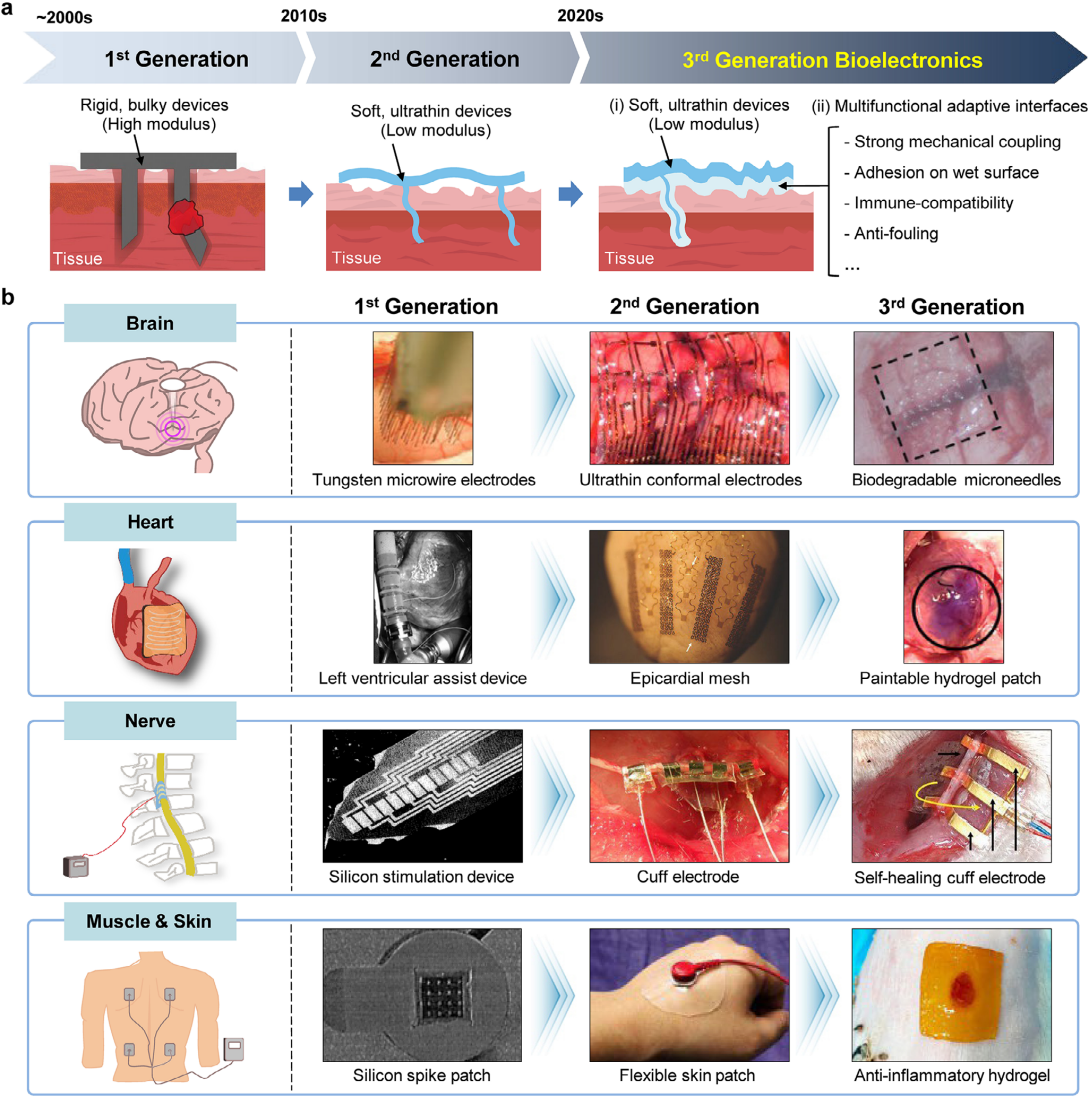
Advances in bioelectronic technologies. (a) Generational characteristics of bioelectronics devices. (b) Evolution of bioelectronic technologies across various human organs, including the brain, heart, nerve, muscle, and skin. (Brain); Reproduced with permission [12]. Copyright 2003, National Academy of Sciences. Reproduced with permission [19]. Copyright 2010, Springer Nature. Reproduced with permission [7]. Copyright 2021, John Wiley and Sons. (Heart); Reproduced with permission [13]. Copyright 2005, Elsevier. Reproduced with permission [20]. Copyright 2015, John Wiley and Sons. Reproduced with permission [4]. Copyright 2024, Elsevier. (Nerve); Reproduced with permission [14]. Copyright 1991, IEEE. Reproduced with permission [21]. Copyright 2010, Elsevier. Reproduced with permission [5]. Copyright 2021, John Wiley and Sons. (Muscle & Skin); Reproduced with permission [15]. Copyright 2001, IEEE. Reproduced with permission [22]. Copyright 2011, John Wiley and Sons. Reproduced with permission [6]. Copyright 2022, American Chemical Society.

**FIGURE 3:**
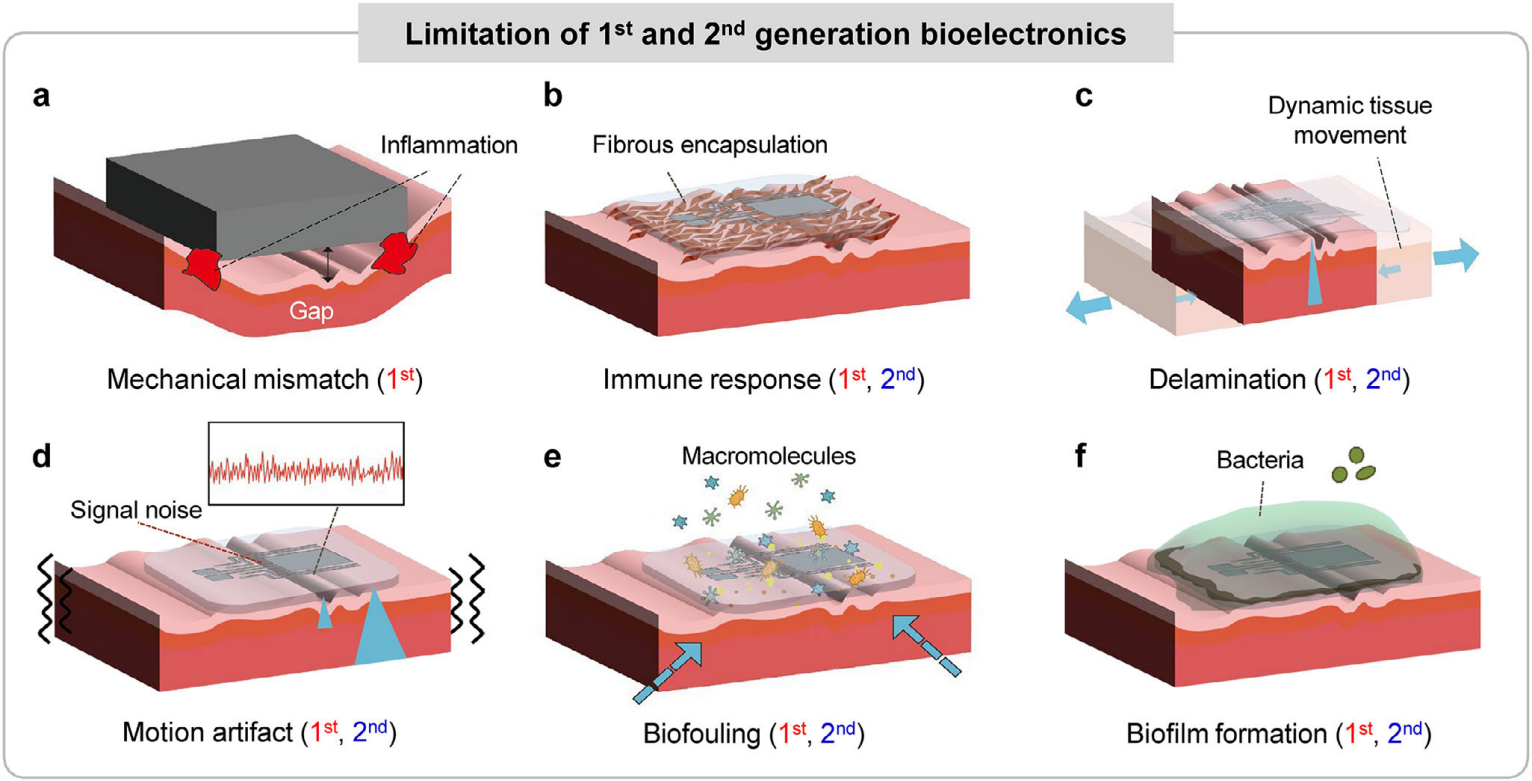
Limitations of first and second generation bioelectronics. (a) Mechanical mismatch between rigid device and soft tissue. (b) Fibrous encapsulation induced by the immune response. (c) Delamination induced by dynamic tissue movement. (d) Signal noise induced by motion artifact. (e) Biofouling issues induced by macromolecules. (f) Biofilm formation induced by interaction with bacteria.

The technological evolution of bioelectronic systems can be broadly classified into three generations according to their device architectures and tissue‐interfacing strategies (Figure 2a,b). Early, first‐generation platforms were predominantly fabricated from rigid materials such as silicon and metals, resulting in stiff electrodes and bulky chip‐based systems. Representative examples from the early 2000s include metal microwire arrays for neural recording and stimulation, as well as implantable cardiac devices, such as ventricular assist systems, constructed with substantial metallic components [12, 13, 14, 15]. Although these technologies established the foundation for clinical bioelectronics, their inherent rigidity imposed critical constraints on long‐term functionality [16]. Stiff electrodes are unable to conform to the soft, curved topography of biological tissues, often creating interfacial air gaps that degrade recording fidelity and stimulation efficiency (Figure 3a) [17]. In addition, the significant mechanical mismatch between rigid devices and compliant tissues induces chronic micromotion at the interface, provoking inflammatory responses and ultimately leading to fibrotic encapsulation, which further compromises signal stability and long‐term device performance (Figure 3b) [18].

To overcome the limitations of rigid bioelectronic platforms, research progressed toward second‐generation systems designed to emulate the mechanical properties of soft biological tissues. Early strategies focused on reducing the thickness of electronic materials to lower flexural rigidity or employing strain‐accommodating structural motifs, such as serpentine interconnects and buckled geometries, to achieve mechanical stretchability [19, 20, 21, 22]. A notable milestone was the epidermal electronic system reported by Kim et al. in 2011, which integrated ultrathin silicon nanomembranes and serpentine interconnects onto a skin‐like substrate to realize conformal, multifunctional physiological sensing [23]. More recent developments have shifted from structural adaptations to the incorporation of intrinsically soft electronic materials that provide inherent stretchability and electrical conductivity without relying solely on mechanical design. These materials are typically engineered as electronic composites in which low‐dimensional functional fillers‐such as metallic nanomaterials, carbon‐based allotropes, or two‐dimensional nanoflakes‐as well as conducting polymers or ionic conductors, are incorporated into a soft elastomeric matrix [24, 25, 26, 27].

Although second‐generation bioelectronic systems have markedly improved sensing and stimulation performance through their ability to establish conformal contact with curved tissue surfaces, several critical challenges continue to impede their clinical translation [28]. Foremost, the dynamic motion of tissues and surrounding organs can destabilize the tissue‐device interface; without reliable fixation, devices may delaminate or generate substantial motion artifacts during physiological movement, thereby degrading signal fidelity. While conventional fixation methods, such as suturing, can be applied, they carry risks such as bleeding and are unsuitable for fragile organs, including the brain and heart (Figure 3c,d). In parallel, the complex biochemical environment within the body introduces additional barriers to long‐term stability. Biofouling and biofilm formation, which arise from the accumulation of macromolecules, bacteria, and corrosive ions on device surfaces, can progressively impair electrical performance and structural integrity (Figure 3e,f) [29, 30]. For implantable systems, these degradation pathways often necessitate device removal after limited operational periods, imposing substantial physical and psychological burdens on patients.

Since the early 2020s, research has increasingly shifted toward third‐generation bioelectronics aimed at addressing the persistent challenges of long‐term stability and reliability. These next‐generation systems not only employ intrinsically soft electronic materials but also integrate engineered interfaces capable of adaptive, multifunctional interactions with human tissues. In this context, we define an adaptive interface as a bioelectronic interface that actively or dynamically adjusts its mechanical, structural, chemical, or physiological interactions with the surrounding biological environment to maintain stable coupling and long‐term functionality. Interfaces that merely provide passive mechanical softness without such adaptive interactions are therefore categorized as second‐generation bioelectronics rather than adaptive biointerfaces. Broadly, these adaptive interfaces can be classified into mechano‐adaptive and biophysiologically adaptive modalities.

**FIGURE 4:**
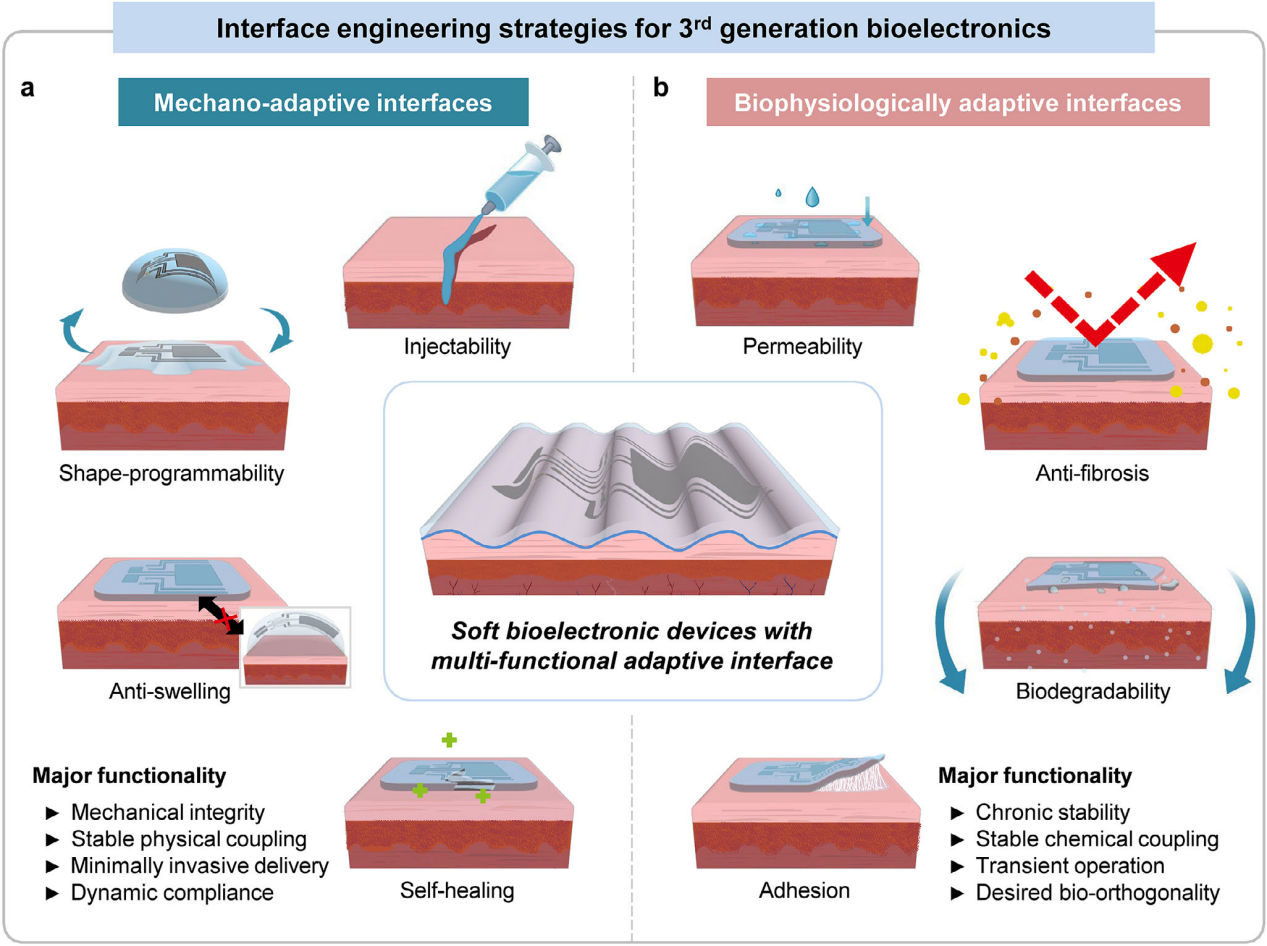
Third‐generation bioelectronics with multi‐functional adaptive interface. (a) Characteristics of mechano‐adaptive interfaces, including injectability, shape‐programmability, anti‐swelling, and self‐healing. (b) Characteristics of biophysiologically adaptive interfaces, including permeability, anti‐fibrosis, biodegradability, and adhesion.

Mechano‐adaptive interfaces are designed to provide dynamic mechanical compliance, structural robustness, and stable physical coupling through advanced structural and material engineering (Figure 4a). Such systems allow device structures or geometries to conform dynamically to wet, curved, and continuously moving biological surfaces, thereby maintaining intimate contact despite tissue deformation and motion. Examples include shape‐programmable devices that actively conform to tissue surfaces in response to stimuli such as humidity or temperature, as well as self‐healing systems capable of restoring mechanical integrity or electrical conductivity degraded by repetitive tissue‐induced strain. Mechano‐adaptive [31, 32, 33] In contrast, biophysiologically adaptive interfaces aim to maintain bio‐orthogonal function within the chemically complex in vivo environment, thereby supporting chronic operational stability (Figure 4b). These interfaces are designed to operate reliably despite biological responses at the tissue‐device interface, including fibrosis, immune reactions, and dynamic biochemical changes in the surrounding microenvironment. Anti‐fouling surfaces minimize macromolecular accumulation and mitigate biofilm formation, while bioadhesive interfaces facilitate reliable, noninvasive attachment to tissues, ensuring sustained high‐quality sensing and stimulation without the need for suturing or other invasive fixation methods [34, 35, 36].

In this review, we summarize recent advances in multifunctional adaptive interfaces that underpin the emergence of third‐generation bioelectronics. We begin by discussing the technologies for mechano‐adaptive interfaces, including shape programmability, injectability, anti‐swelling characteristics, and self‐healing mechanisms, followed by strategies for biophysiologically adaptive interfaces such as controlled permeability, anti‐fibrotic design, tissue adhesion, and biodegradability. We then highlight representative wearable and implantable bioelectronic systems that integrate these adaptive interfaces and conclude with a perspective on future opportunities and challenges for translating these technologies toward long‐term, clinically viable human machine interfaces.

## 2 Adaptive Interfaces for Third Generation Bioelectronics

While second‐generation bioelectronics substantially reduced modulus mismatch through soft materials and stretchable architectures, limitations in long‐term stability, interfacial reliability, and in vivo performance have driven the emergence of more functionally integrated interface designs. Third‐generation bioelectronic interfaces therefore, extend beyond passive mechanical compliance and incorporate adaptive interfacial functionalities that actively respond to dynamic mechanical, chemical, and biological environments.

In this section, we examine recent advances in adaptive interface engineering that aim to improve mechanical robustness, bio‐interfacial stability, and chronic operational reliability. Such approaches could be organized into two categories: mechano‐adaptivity, encompassing physical strategies that enable devices to accommodate dynamic tissue motion, and biophysiological adaptivity, encompassing chemical strategies that ensure stable operation within the complex in vivo environment. In this chapter, we highlight recent advances that impart these adaptive functionalities to third‐generation bioelectronic interfaces, showcasing representative examples that illustrate their expanding capabilities.

### 2.1 Mechano‐Adaptive Bioelectronic Interfaces

The seamless integration of artificial electronic systems with biological tissues requires precise mechanical and functional matching across inherently soft, dynamic, and heterogeneous interfaces. Although earlier generations of bioelectronics largely focused on reducing modulus mismatch between rigid devices and compliant tissues [37, 38], the complex and continuous motions present in vivo introduce challenges that extend far beyond static mechanical compatibility. Biological environments undergo diverse deformation patterns, ranging from subtle neural micromotions to the rhythmic expansion of cardiac muscle and the multidirectional strains of the skin, each with distinct amplitudes and frequencies [39, 40]. For long‐term, stable operation, artificial systems must accommodate these irregular geometries and dynamic mechanical loads while minimizing surgical invasiveness. Accordingly, next‐generation soft human‐machine interfaces (HMIs) require mechano‐adaptivity: the capacity to dynamically conform to tissue surfaces, maintain intimate coupling, and preserve functional performance under complex and evolving mechanical stimuli.

Recent progress in materials science and microfabrication has yielded a suite of effective strategies to impart mechano‐adaptivity to soft HMIs (Table 1). Previously reported mechano‐adaptive interfaces include four representative modalities: shape‐programmability, injectability, anti‐swelling characteristics, and self‐healing capability. Geometry‐guided architectures incorporating actuators or stimuli‐responsive materials enable pre‐programmed deformation modes that accommodate curvilinear or dynamically moving organs, thereby achieving conformal contact and stable device operation over extended periods [41, 42, 43, 44, 45, 46]. Injectable and in situ formable platforms reduce surgical invasiveness and facilitate adaptive integration by enabling minimally invasive delivery and localized formation of compliant interfaces within confined or irregular biological environments [47, 48, 49, 50, 51]. Anti‐swelling hydrogel matrices maintain dimensional stability and electrical integrity in aqueous or ionic environments, preventing modulus drift, delamination, and signal degradation over time, which is an essential requirement for both wearable and implantable operations [52, 53, 54, 55]. In parallel, self‐healing materials counteract mechanical fatigue, microcrack formation, and long‐term structural damage by autonomously restoring mechanical and electrical continuity during repeated deformation or chronic implantation, thereby prolonging device lifetime and enhancing reliability [56, 57, 58, 59]. Collectively, these strategies shift soft HMI design from passive flexibility toward active mechanical adaptability, redefining how electronic interfaces can dynamically engage with living systems.

**TABLE 1:** Recent studies on third‐generation bioelectronics with mechano‐adaptive interfaces.

Tissue	Year	Device	Modulus	Mechano‐adaptivity				Refs.
				Shape‐programmability	Injectability	Anti‐swelling	Self‐healing	
Brain	2017	SiNW/SU‐8 mesh	—	Elastic unfolding	Geometric self‐restoration	—	—	[47]
	2023	PEG‐SH/PEG‐VS hydrogel	—	—	In‐situ click crosslinking	High crosslinking density (Swelling ratio: ∼ 1.33)	—	[60]
	2024	SMP/Parylene‐C ECoG array	—	Shape memory; thermally‐triggered reconfiguration; pre‐stored strain	Thermally‐triggered unfolding	—	—	[41]
	2024	Fluidic‐actuated PDMS/Au ECoG	—	Fluidic pressure‐triggered actuation	Fluidic pressure‐triggered actuation	—	—	[42]
	2025	PHEMA‐PPEGMA nanofibrous hydrogel	—	—	Shear‐thinning	—	Intrinsic; jammed fibrous network	[61]
Heart	2023	PAACP hydrogel	40 kPa	—	—	Hydrophobic polymer (Swelling ratio: ∼ 1.2)	—	[52]
	2024	NdFeB‐based PFM	—	—	Flowable magnetic colloid	—	—	[48]
Muscle	2023	IT‐IC hydrogel	G′^b)^: 188.9 ± 28.3 Pa	—	Biphenyl rearrangement & Shear‐thinning	—	Intrinsic; dynamic bonding	[51]
	2024	PVA/CNF/PEDOT:PSS hydrogel	3.71 MPa	—	—	Densification of polymer network (Swelling ratio: < 3%)	—	[62]
	2025	PAAc‐CaAc hydrogel	0.1 MPa	Shape memory; trigger‐free	—	—	—	[63]
Nerve	2020	PDMS‐MPU SHP/Ag composite	163.3 ± 42.1 kPa	—	—	—	Intrinsic; dynamic bonding	[58]
	2023	PEO/PEG‐α‐CD film	Dry: 260 MPa Wet: 100 kPa	Water‐induced contraction (contraction; > 50%)	—	—	—	[43]
	2024	TA‐In‐PVA hydrogel	6.42–40.9 kPa	—	Shear‐thinning	Tannic acid‐based dynamic non‐covalent bonds (Swelling ratio: 9.22% ± 0.76%)	—	[54]
	2024	BSS‐PLCL elastomer	0.19 MPa	Shape memory; triggered phase transition (at ∼38°C)	—	—	—	[64]
Skin	2022	PF127/HA‐MA hydrogel	200 kPa	—	In situ UV crosslinking	Hydrophobic polymer (Swelling ratio: ± 1%)	—	[65]
	2023	HA‐OPA/PEG‐hydrazide hydrogel	G′^b)^: 14.8 kPa	—	In situ hydrazone crosslinking	—	Intrinsic; dynamic hydrazone linkages	[50]
	2025	PBA‐Flavonoid hydrogel	G′^b)^: 350 Pa	—	Shear‐thinning	—	Intrinsic; dynamic borate ester bonds	[66]
Stomach	2025	TCP‐25/CMC‐based hydrogel	—	—	Shear‐thinning	—	Intrinsic; dynamic bonding	[67]
Vein	2021	Oxime‐urethane PU elastomer	172 kPa–3.742 MPa	—	—	—	Intrinsic; dynamic oxime–urethane bonds	[59]
	2024	Needle‐like Biphasic Microfiber (Gallium/Nanocomposite)	Rigid: 9.8 GPa Soft: 30 kPa	Temperature‐triggered phase transition (at 37°C)	Phase transition‐triggered softening	—	—	[44]

#### 2.1.1 Shape‐Programmability

Soft HMIs must conformally integrate with target tissues while providing sufficient spatial coverage for accurate sensing, effective stimulation, and localized therapy. Shape‐programmable systems address this requirement by offering active configurability, enabling devices to morph, deploy, or self‐fit within confined or geometrically complex biological environments. These systems can be broadly categorized into two classes according to their actuation mechanisms: mechanically triggered shape reconfiguration and stimuli‐responsive shape morphing. Both approaches seek to establish stable, minimally invasive, and adaptive bio‐interfaces capable of dynamically conforming to tissue geometry.

Mechanically triggered systems utilize pre‐engineered geometries that incorporate embedded actuators or pre‐strained components capable of storing mechanical energy in a constrained state. Upon activation by an external stimulus, commonly thermal or pneumatic, this stored energy is released, allowing the structure to transition to its deployed configuration. Such mechanisms enable compact, minimally invasive delivery through small incisions, followed by controlled expansion or unfolding to achieve broad, conformal coverage of the targeted tissue surface.

**FIGURE 5:**
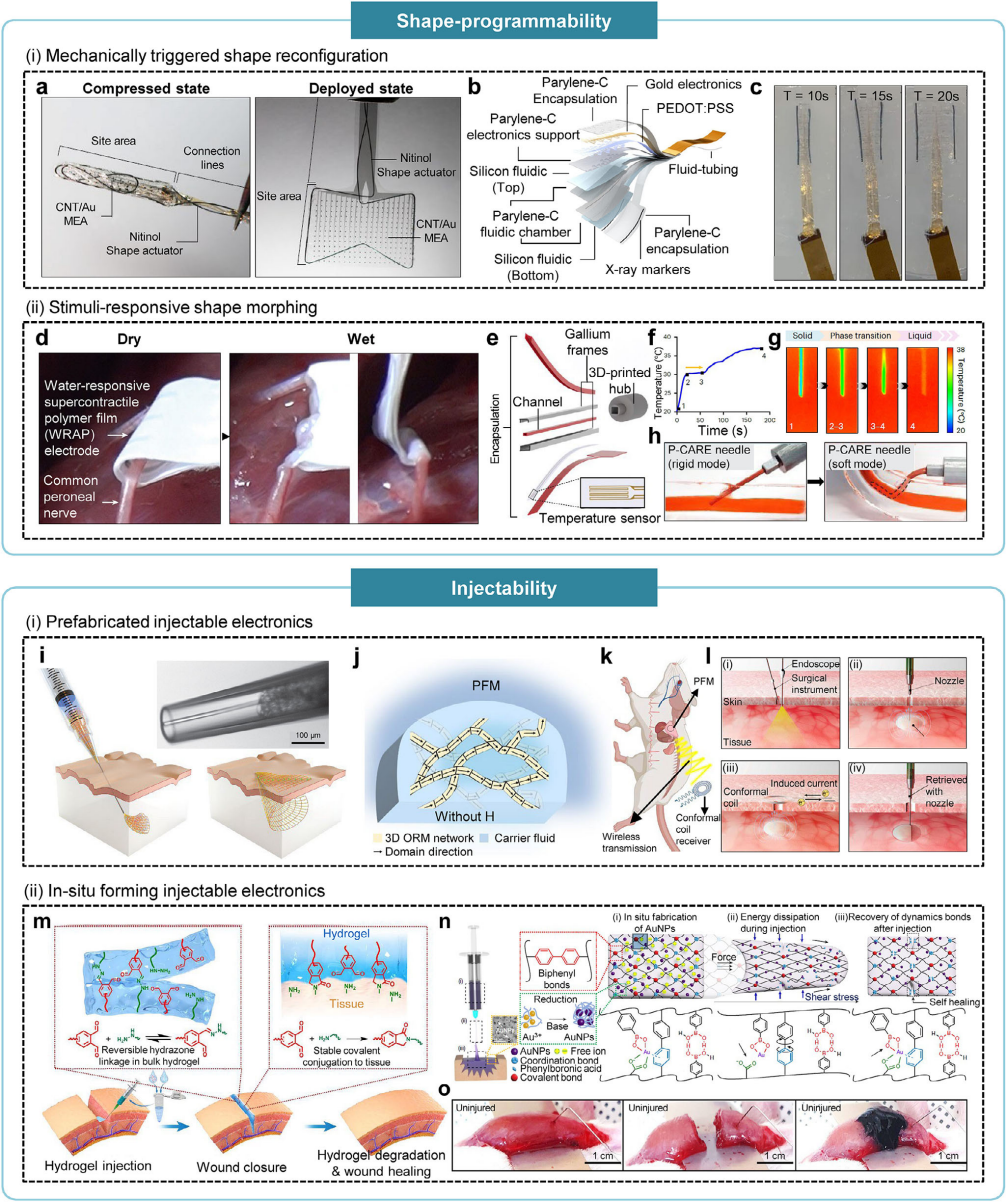
Mechano‐adaptive bioelectronic interface with shape‐programmability and injectability. (a) Optical images of a shape‐changing electrode array in compressed and deployed states. Reproduced with permission [41]. Copyright 2024, Springer Nature. (b) Exploded schematic illustration of the layered structure of an origami‐inspired ECoG system. (c) Optical images showing pneumatic expansion of the origami‐inspired ECoG system from folded to unfolded configurations. Reproduced with permission [42]. Copyright 2024, Springer Nature. (d) Optical images of a water‐responsive WRAP electrode contracting and conformally wrapping around a nerve. Reproduced with permission [43]. Copyright 2023, Springer Nature. (e) Exploded schematic illustration of a temperature‐responsive gallium needle. f) Phase transition profile of the gallium needle under a temperature stimulus of 37°C. (g) Infrared thermal images of the temperature‐responsive needle during phase transition. (h) Optical images of the temperature‐responsive needle in rigid and softened states. Reproduced with permission [44]. Copyright 2024, Springer Nature. (i) Schematic illustration of syringe‐injectable mesh electronics; inset shows a microscopic image of the mesh electronics within a glass needle. Reproduced with permission [47]. Copyright 2015, Springer Nature. (j) Schematic illustration of a PFM showing a 3D oriented magnetic network suspended in carrier fluid. (k) Schematic illustration of PFM‐based bioelectronics for wireless monitoring of internal physiological signals. (l) Schematic illustration showing (i) target determination, (ii) injection, (iii) in situ measurement, and (iv) retrieval of PFM‐based bioelectronics. Reproduced with permission [48]. Copyright 2024, Springer Nature. (m) Schematic illustration of dynamic cross‐linking and tissue adhesion mechanisms in OPA/hydrazide‐based hydrogels and their applications to wound sealing. Reproduced with permission [50]. Copyright 2023, The American Association for the Advancement of Science. (n) Schematic illustration showing injection and in situ formation mechanisms of an injectable prosthesis. (o) Optical images demonstrating muscle treatment using the injectable prosthesis. Reproduced with permission [51]. Copyright 2023, Springer Nature.

For example, Wei et al. developed a shape‐transformable electrode array that enables minimally invasive, large‐area electrocorticography (ECoG) mapping [41]. The ultrathin array, constructed from Au/CNT hybrid conductors on a Parylene‐C substrate, was integrated with a nitinol shape‐memory actuator programmed to recover its expanded configuration at physiological temperature (∼37°C). During implantation, the device was compactly rolled into a narrow strip suitable for insertion through a 2‐mm cranial opening, after which it autonomously redeployed into a wide‐area array conforming to the cortical surface (Figure 5a). This transformation was driven by pre‐stored mechanical strain within the actuator, while a dissolvable polyethylene oxide adhesive modulated the unfolding rate to ensure controlled and predictable deployment. Such geometry‐driven reconfiguration enabled neural mapping across large cortical regions while maintaining minimal surgical invasiveness.

Similarly, Coles et al. reported an origami‐inspired soft fluidic actuator designed for large‐area ECoG applications [42]. The system incorporated parylene‐C microfluidic chambers embedded within a PDMS encapsulation layer, enabling controlled pressurization and expansion without fluid leakage during actuation (Figure 5b). In its folded configuration, the electrode sheet could be inserted subdurally through a small burr‐hole craniotomy (∼6 mm) and subsequently expanded by internal air pressure to achieve wide cortical coverage (Figure 5c). Leveraging soft‐robotic actuation principles, the internally generated pressure facilitated predictable unfolding and conformal deployment along the brain surface. By integrating flexible electronics with soft‐fluidic actuation, this platform presented a clinically practical strategy for minimally invasive, large‐area neural recording.

Stimuli‐responsive systems enable shape transformation through intrinsic material responses to external cues such as moisture, temperature, light, or magnetic fields. These materials autonomously adjust their geometry in accordance with environmental changes, allowing self‐adaptive deployment and sustained conformality following implantation. Yi et al. introduced water‐responsive supercontractile polymer films (WRAP) as a bioadaptive interface material inspired by the supercontraction behavior of spider silk [43]. The WRAP films, composed of polyethylene oxide and PEGα‐cyclodextrin inclusion complexes, underwent more than 50% contraction upon hydration, transitioning from a dry, mechanically stiff state suitable for electronic integration to a soft, hydrogel‐like state with a modulus of approximately 100 kPa. This hydration‐induced contraction enabled electrode‐integrated WRAP films to conformally wrap around nerves, muscles, and cardiac tissues, supporting high‐fidelity electrophysiological recording and stimulation (Figure 5d). The rapid, biocompatible, and externally benign water‐triggered actuation underscored the potential of WRAP films as a versatile platform for self‐adaptive soft bioelectronic interfaces.

In another work, Agno et al. introduced a temperature‐responsive intravenous needle that irreversibly softens upon exposure to physiological conditions. The device incorporated a gallium‐based metallic frame encapsulated within an elastomeric matrix. (Figure 5e) [44]. While the gallium structure remained rigid at room temperature to facilitate insertion, it melted near 30°C, transitioning the device into a soft and compliant form that readily conforms to surrounding tissues (Figure 5f–h). This phase‐transition‐driven stiffness modulation reduced tissue trauma and mitigated needlestick‐related risks while preserving mechanical stability and effective fluid delivery. Such temperature‐triggered shape morphing offered a practical and clinically relevant strategy for achieving dynamic mechanical adaptability in minimally invasive biomedical tools.

#### 2.1.2 Injectability

While shape‐programmable architectures can achieve conformal contact through controlled structural reconfiguration, their implantation generally necessitates surgical access. To further reduce invasiveness and enable integration within irregular or deep tissue environments, injectability offers a complementary strategy in which flowable devices or material precursors are delivered through small openings and subsequently reconstitute in situ into stable, functional interfaces. Based on their formation mechanisms, injectable systems can be broadly categorized into two classes: prefabricated injectable electronics and in situ‐forming injectable materials.

Prefabricated injectable electronics achieve injectability through mechanical or colloidal engineering strategies that allow fully assembled devices to traverse narrow conduits and subsequently recover their architecture and functional performance after delivery. Liu et al. demonstrated this concept using syringe‐injectable electronics constructed as a centimeter‐scale, microporous ultrathin mesh in which the unit‐cell geometry modulates longitudinal and transverse bending stiffness [47]. This design enabled the mesh to be tightly rolled without fracture and pass through glass needles with an inner diameter (ID) as small as 95 µm, after which it elastically returned to its pre‐patterned two‐dimensional configuration upon ejection (Figure 5i). Device yield and post‐injection electrical metrics, including impedance and conductance, were systematically evaluated across needle gauges, confirming robust structural and functional recovery. Injectability in this system was governed by geometric mechanics; specifically, the optimized bending stiffness of an ultrathin lattice, which allowed a solid electronic network to be delivered through fine needles and re‐establish stable, chronic interfaces following deployment.

A complementary liquid‐state approach can be exemplified by permanent fluidic magnets (PFMs). Zhao et al. developed PFMs by dispersing non‐Brownian neodymiumironboron (NdFeB) nanomagnets within a viscous silicone carrier and applying impulse magnetic fields to induce the self‐assembly of a three‐dimensional, oriented‐and‐ramified magnetic network (Figure 5j) [48]. This engineered colloidal architecture decoupled Brownian relaxation from colloidal stability, producing a permanent magnetic liquid that remained injectable while exhibiting sustained remanent magnetization and coercivity. Following needle‐based delivery, the stable magnetic flux of the injected PFM inductively coupled with an external conformal coil, enabling self‐powered wireless cardiovascular monitoring, and the PFM could be retracted from the target site using a needle (Figure 5k,l).

In situ‐forming injectable materials can be delivered as shear‐thinning precursors that rapidly solidify within the body through covalent crosslinking or noncovalent interactions such as hydrogen bonding, electrostatic association, or ππ stacking. Ren et al. demonstrated this concept using injectable, self‐healing hydrogel adhesives formed via catalyst‐free o‐phthalaldehyde (OPA)/hydrazide condensation, which generates hydrazone‐crosslinked hyaluronic acid (HA)/PEG hydrogels under physiological conditions without initiators or UV irradiation and produces only water as a by‐product [50]. Residual OPA groups react spontaneously with tissue amines to form stable phthalimidine linkages, enabling strong wet‐tissue adhesion (Figure 5m); for instance, 7 wt% OPA/hydrazide HAPEG hydrogels achieved an adhesion strength of 27.6 ± 3.9 kPa. The dynamic hydrazone network also imparted self‐healing and energy‐dissipative characteristics, supporting sutureless wound sealing and conformal adhesion to a variety of tissues following injection.

Building on similar dynamic‐bonding principles, Jin et al. developed an injectable tissue prosthesis based on a phenylborate‐mediated hydrogel. The material formed a soft, electroactive network through multiple crosslinking modes, including irreversible yet rearrangeable biphenyl covalent bonds and reversible coordination interactions (Figure 5n). Concurrent in situ reduction of Au(III) yielded embedded gold nanoparticles, imparting immediate bidirectional electrical conductivity throughout the matrix. Rheological characterization showed that the optimized formulation rapidly transitioned into a hydrogel with a high storage modulus (188.9 ± 28.3 Pa at 1 Hz) and low loss factor (0.018 ± 0.003) within one minute, supporting shear‐thinning flow through fine needles and fast structural recovery after injection. Functionally, the injectable prosthesis could fill irregular defects, bridge neuromuscular circuits, and enable instantaneous closed‐loop rehabilitation by combining conformal volumetric filling with stable, conductive interfacing (Figure 5o).

#### 2.1.3 Anti‐Swelling

Soft HMIs operating in aqueous and ionic environments inevitably absorb water, and uncontrolled swelling can increase device thickness, aggravate modulus mismatch, generate interfacial shear, weaken wet adhesion, and disrupt electrical percolation pathways, which ultimately compromise signal fidelity and long‐term stability. To address these challenges, anti‐swelling strategies are essential to restrict bulk water uptake while preserving adhesion strength, mechanical robustness, and electrochemical performance.

**FIGURE 6:**
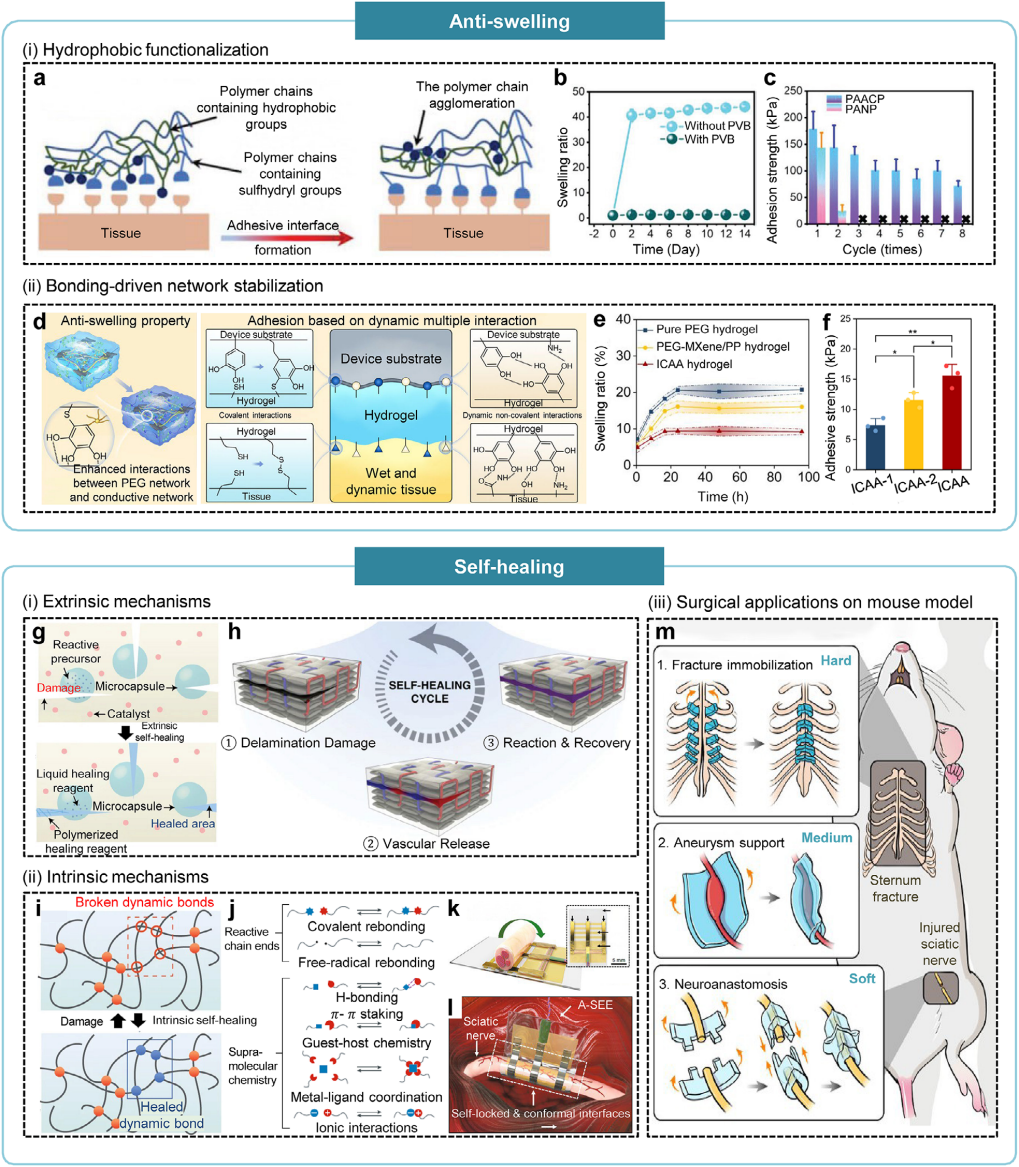
Mechano‐adaptive bioelectronic interface with anti‐swelling and self‐healing properties. (a) Schematic illustration of polymer chains containing hydrophobic and sulfhydryl groups. (b) Swelling ratios of hydrogels with and without PVB over time. (c) Adhesion strengths of PVB‐based hydrogel (PAACP) and its counterpart (PANP) under repeated adhesion cycles. Reproduced with permission [52]. Copyright 2023, John Wiley and Sons. (d) Schematic illustration of anti‐swelling hydrogels featuring enhanced dynamic and multivalent interactions. (e) Swelling ratios of hydrogels with varying compositions. (f) Adhesion strengths of anti‐swelling hydrogels with different solid contents. Reproduced with permission [54]. Copyright 2024, Springer Nature. Schematic illustrations of extrinsic self‐healing mechanisms (g) using microcapsule and (h) vascular networks. Reproduced with permission [56] Copyright 2019, Springer Nature. Reproduced with permission [68]. Copyright 2014, John Wiley and Sons. (i) Schematic illustration of intrinsic self‐healing mechanisms based on reversible dynamic bonding. Reproduced with permission [56]. Copyright 2019, Springer Nature. (j) Representative types of covalent and noncovalent interactions involved in intrinsic self‐healing. Reproduced with permission [57]. Copyright 2020, Springer Nature. Schematic illustrations showing the structure of (k) a self‐locking A‐SEE and (l) its conformal integration around a sciatic nerve. Reproduced with permission [58]. Copyright 2020, Springer Nature. (m) Schematic illustration of three representative biomedical applications of self‐healing elastomers, categorized according to their mechanical properties. Reproduced with permission [59]. Copyright 2021, Springer Nature.

Tian et al. developed a nonswelling, tissue‐adhesive hydrogel by physically incorporating hydrophobic poly(vinyl butyral) (PVB) into an acrylic acid/gelatin/chitosan‐N‐acetyl‐L‐cysteine network [52]. In contrast to conventional approaches that rely on increasing crosslinking density or covalently grafting hydrophobic moieties, PVB was introduced without crosslinking or copolymerization, forming dispersed hydrophobic domains that effectively hinder water penetration while preserving the mobility of adhesive functional groups (Figure 6a). This design yielded a hydrogel with a low swelling ratio of 1.2 and exceptionally strong wet‐tissue adhesion (peak strength 211.4 kPa), nearly an order of magnitude higher than prior nonswelling formulations (Figure 6b,c). Adhesion strength remained above 70 kPa after eight attach‐detach cycles, enabled by reversible disulfide (SS) bonding at the tissue interface. Moreover, the nonswelling matrix suppressed chronic inflammation and maintained stable electrode‐tissue coupling under cyclic deformation, demonstrating that physically engineered hydrophobic barriers can effectively limit swelling without compromising adhesion, biocompatibility, or long‐term bioelectronic performance.

In contrast, Yang et al. achieved anti‐swelling behavior through network stabilization rather than hydrophobic barrier formation [54]. They engineered a hydrogel that was simultaneously anti‐swelling, injectable, and conductive by integrating a thiolmaleimide PEG network with a multiscale conductive architecture (MXene/PEDOT:PSS) and employing tannic acid (TA) as a molecular regulator. TA moderated the click‐reaction kinetics to preserve injectability while establishing dense hydrogen bonding and ππ interactions between the PEG matrix and conductive phases, thereby imparting anti‐swelling properties and enhancing electrical conductivity (Figure 6d). The optimized formulation exhibited a low swelling ratio of 9.22% ± 0.76% and improved adhesion strength (15.6 ± 1.9 kPa), along with long‐term electrochemical stability (Figure 6e,f). Functionally, the hydrogel operated as a form‐fitting cuff electrode for chronic vagus nerve neuromodulation, maintaining consistent performance over extended implantation. The anti‐swelling capability arose from synergistic covalent and noncovalent interactions that increased network cohesion and crosslink density without compromising softness, injectability, or conformal tissue interfacing.

#### 2.1.4 Self‐Healing

Soft HMIs inevitably experience mechanical damage such as microcrack formation, interfacial delamination, and fatigue accumulation during cyclic deformation or long‐term implantation. Such structural defects disrupt electrical percolation pathways and impair mechanical compliance, ultimately accelerating device degradation and failure. To enhance operational lifetime and ensure stable functionality, self‐healing materials have been introduced to autonomously restore structural and electrical integrity through molecular‐ or chemistry‐driven repair processes. Depending on their underlying healing mechanisms, these strategies can be broadly classified into extrinsic and intrinsic self‐healing modalities.

Extrinsic self‐healing systems incorporate healing agents within microcapsules, vascular networks, or nanoparticles dispersed throughout the matrix. When cracks propagate, these reservoirs rupture and release the encapsulated agents, which wet the fracture surfaces and subsequently polymerize to restore structural continuity (Figure 6g,h) [56, 68, 69, 70]. Although this approach can achieve high healing efficiency, it presents several limitations for soft biointerfaces: the repair is generally non‐repeatable once the stored agents are depleted; embedded capsules or vascular channels can alter the composite's softness and mechanical compliance; and many healing chemistries involve agents that may be cytotoxic or incompatible with physiological environments. Additionally, the polymerization kinetics and restricted mobility of released agents hinder effective repair in dynamic, hydrated tissue environments. As a result, extrinsic self‐healing strategies are largely unsuitable for long‐term, compliant bioelectronic systems, where repeated, biocompatible, and mechanically unobtrusive repair is essential.

In contrast, intrinsic self‐healing strategies incorporate reversible dynamic bonds directly into the polymer backbone, enabling continuous bond exchange and network reformation without the need for external healing agents (Figure 6i) [56]. These dynamic interactions span a broad range of reversible chemistries, including covalent mechanisms such as imine exchange, disulfide reshuffling, boronate ester formation, and Diels‐Alder reactions, as well as noncovalent interactions such as hydrogen bonding, ionic or metalligand coordination, ππ stacking, and van der Waals forces (Figure 6j) [57]. The autonomous reformation of these reversible bonds supports repeated healing under physiological conditions while preserving the mechanical softness, elasticity, and electrical conductivity required for stable bioelectronic interfacing.

For example, Song et al. developed a dynamically crosslinked self‐healing polymer (A‐SEE) incorporating multivalent reversible bonds that imparted dynamic stress relaxation, waterproof sealing, and autonomous self‐bonding [58]. The material self‐locked around the sciatic nerve without the need for sutures or adhesives, achieving conformal wrapping and low interfacial stress during body motion (Figure 6k,l). Continuous bond exchange within the reversible crosslink network preserved both electrical conductivity and mechanical compliance throughout chronic implantation. In vivo, this adaptive, self‐healing electronic epineurium enabled stable bidirectional neural recording and stimulation, including nerve‐to‐nerve feedback signaling, demonstrating how intrinsic self‐healing mechanisms can enhance device longevity, functional reliability, and biological integration.

Similarly, Jiang et al. developed a self‐healing polyurethane elastomer based on dynamic oximeurethane exchange and hydrogen bonding [59]. By modulating the crosslinking density, the mechanical properties could be precisely tuned, with tensile strength ranging from 33 kPa to 4.383 MPa and Young's modulus from 172 kPa to 3.724 MPa, which falls within the requirements of diverse biomedical environments. Softer formulations in the kilopascal range enabled suture‐free nerve coaptation through autonomous self‐healing, whereas stiffer variants in the megapascal range provided structural reinforcement for vascular or skeletal stabilization, including aneurysm wrapping and sternum immobilization (Figure 6m). This broad mechanical tunability highlighted the versatility of intrinsically self‐healing polymers for targeted, application‐specific biomedical functions.

### 2.2 Biophysiologically Adaptive Bioelectronic Interfaces

Although mechano‐adaptive interfaces effectively mitigate issues arising from modulus mismatch and dynamic tissue deformation, these strategies alone are insufficient to guarantee chronic stability within the living body [71]. Biological tissues constitute a biochemically and immunologically active milieu rich in proteins, lipids, enzymes, reactive oxygen species, and diverse inflammatory mediators. These factors continuously interact with implanted systems, driving biofouling, hydrolysis, enzymatic degradation, and fibrotic encapsulation‐processes that progressively elevate interfacial impedance and erode electrochemical performance, even in soft and conformal devices. Without appropriate biophysiological management, long‐term implantation ultimately results in delamination, instability, and loss of sensing or stimulation fidelity.

Biophysiologically adaptive interfaces directly address these challenges by engineering chemical robustness, immune modulation, and biological integration at the tissue‐device boundary (Table 2). Such strategies regulate mass transport through controlled permeability to gases, water, and ions; suppress fibrotic and inflammatory cascades; and establish strong, durable wet adhesion without invasive fixation [72]. Complementarily, biodegradable materials offer a path toward transient systems that are resorbed after the therapeutic window, thereby eliminating chronic foreign‐body burden. In all, these unconventional biophysiological adaptations enable persistent low‐impedance coupling, stable mechanical integration, and high‐fidelity operation within the biochemically harsh in vivo environment, providing a foundation for reliable chronic use and facilitating clinical translation of next‐generation soft bioelectronic systems.

**TABLE 2:** Recent studies on third generation bioelectronics with biophysiologically adaptive interfaces.

Tissue	Year	Device	Modulus	Biophysiological adaptivity					Refs.
				Permeability	Anti‐fibrosis mechanism	Adhesion strength /interfacial toughness	Tissue‐adhesion mechanism	Biodegradability	
Brain	2010	Silk‐supported Au/PI electrodes	—	—	—	—	Capillary force‐driven adhesion	∼14 days	[19]
	2016	Bioresorbable Si‐NMs/Mg sensors	—	—	Material bioresorbability‐mediated irritation‐free	—	Surgical glue (Fibrin glue/TISSEAL) and sutures	∼3 days	[73]
Heart.	2019	PAAc‐NHS/Gelatin DST	2.5–5 kPa	—	Interfacial water removal‐mediated rapid integration	160 kPa	Interfacial water removal‐mediated covalent crosslinking (NHS‐Amine)	7–28+ days	[74]
	2022	o‐HA‐Tyr/CMC hydrogel	2 kPa	—	Natural polymer bioresorbability‐mediated irritation‐free	20 kPa	Schiff‐base covalent bonding (Aldehyde‐Amine) & Physical entanglement	∼7 days	[75]
	2023	PLCL‐based cardiac jacket	0.05–2 MPa	—	—	3 MPa	Stingray barb‐inspired interlocking mechanism (Mechanical friction)	∼210 days	[76]
	2024	PAAc‐NHS/PVA IPN adhesive	33–63 kPa	—	Conformal integration	—	Covalent (NHS‐Amine) & Physical (H‐bond) IPN crosslinking	—	[77]
	2024	GelMA/ACA AuxES patch	1–20 kPa	—	Drug‐mediated suppression	∼40–50 kPa	Interfacial water absorption‐mediated hydrogen & ionic bonding	∼7 days	[78]
Nerve	2018	PLGA/Mg/Mo Bioresorbable Wireless Stimulator	—	—	Complete material resorption‐mediated irritation‐free	—	Surgical sutures (Nerve cuff integration)	∼25 days	[79]
Skin	2017	PVA‐assisted Au nanomesh	—	WVTR^c)^: ∼100% loss/48h	Nanomesh structure‐mediated high gas permeability‐mediated irritation‐free	—	Conformal contact‐mediated Van der Waals forces	∼Instant	[80]
	2017	s‐PUA based OIA patch	—	—	—	∼40 kPa	Capillary‐assisted suction stress and vacuum state formation	—	[81]
	2018	pNIPAM/PEDOT:PSS/CNT on PDMS adhesive	—	—	—	13.4 kPa	Temperature‐responsive suction effect	—	[82]
	2020	AgNW/porous TPU electrode	—	WVTR^c)^: 23 mg/cm^2^/h; Pore: ∼5 µm	high gas/sweat permeability‐mediated irritation‐free	—	Conformal contact‐mediated Van der Waals forces	—	[83]
	2021	Ultrathin functionalized PAAm hydrogel	8 kPa	O_2_ mass‐permeability; PAAm network	—	—	Conformal contact with rugged skin surface	—	[84]
	2021	P(AN‐co‐AA‐co‐UM) hydrogel	—	—	—	40 kPa	Tree‐frog‐inspired hexagonal micropillar drainage & Shape memory	—	[85]
	2022	TA‐based Interfacial Molecular Lock	—	—	—	11 kPa	Mussel‐inspired catechol‐mediated dynamic covalent/ noncovalent bonding	—	[86]
	2024	3D integrated LM‐fiber e‐skin	0.1–0.31 MPa	Air: 177 mm/s Moisture: 676 g/m^2^/d	Fibrous porous network‐mediated irritation‐free	—	Conformal contact with textile‐like softness	—	[87]
	2024	p(g2T‐T) based Soft semiconducting hydrogel	81.9 kPa	Interpenetrated double network (Size: 20 kDa PEG‐FITC/24h)	—	—	Conformal integration with soft tissue	—	[88]
	2025	LCE‐based Self‐compliant Ionic Nanomesh	326 kPa	WVTR^c)^: >1200 g/m^2^/d; Air: 471 L/m^2^/s	—	370 J/m^2^	LCE‐mediated liquid‐like deformation and self‐adaptive compliance	—	[89]
Stomach	2023	PAASP hydrogel	15 kPa	—	Amino acid‐based biocompatible interface reducing foreign body response	106–120 kPa	Synergetic interfacial hydrogen bonding and high cohesive energy	—	[90]
Subcutaneous	2012	Silk/Mg/Si‐NMs Transient Electronics	—	—	Programmable material resorption	—	Conformal contact‐mediated Van der Waals forces	∼15 days	[91]
	2020	Tough Triazole‐Zwitterionic hydrogel	—	—	Super‐low biofouling interface‐mediated inhibition of adsorption	—	In‐situ encapsulation and physical integration	—	[92]
	2021	Poly‐DL‐serine hydrogel	0.046–0.12 Mpa	—	Bio‐inspired amino acid‐mediated mitigation of cytokine	—	Physical integration with minimal collagen encapsulation	∼210 days	[93]
	2024	Zwitterion‐albumin hybrid hydrogel	10–80 kPa	—	Hybrid‐network mediated antifouling	—	Physical integration and stable tissue‐conformal contact	∼90 days	[94]
	2025	Immune‐compatible p(g2T‐Se)‐TMO OECT	—	—	formation of a hydration layer‐mediated anti‐biofouling	—	Stable interfacial integration with suppressed FBR	∼90 days	[95]
Vein	2021	Trigger‐detachable Boronate‐hydrogel	G′^b)^: 2.5 kPa	—	On‐demand detachment‐mediated prevention of post‐procedural trauma	>400 J/m^2^	Dynamic boronate‐diol complexation & Hydrogen bonding	∼28 days	[96]

#### 2.2.1 Permeability

**FIGURE 7:**
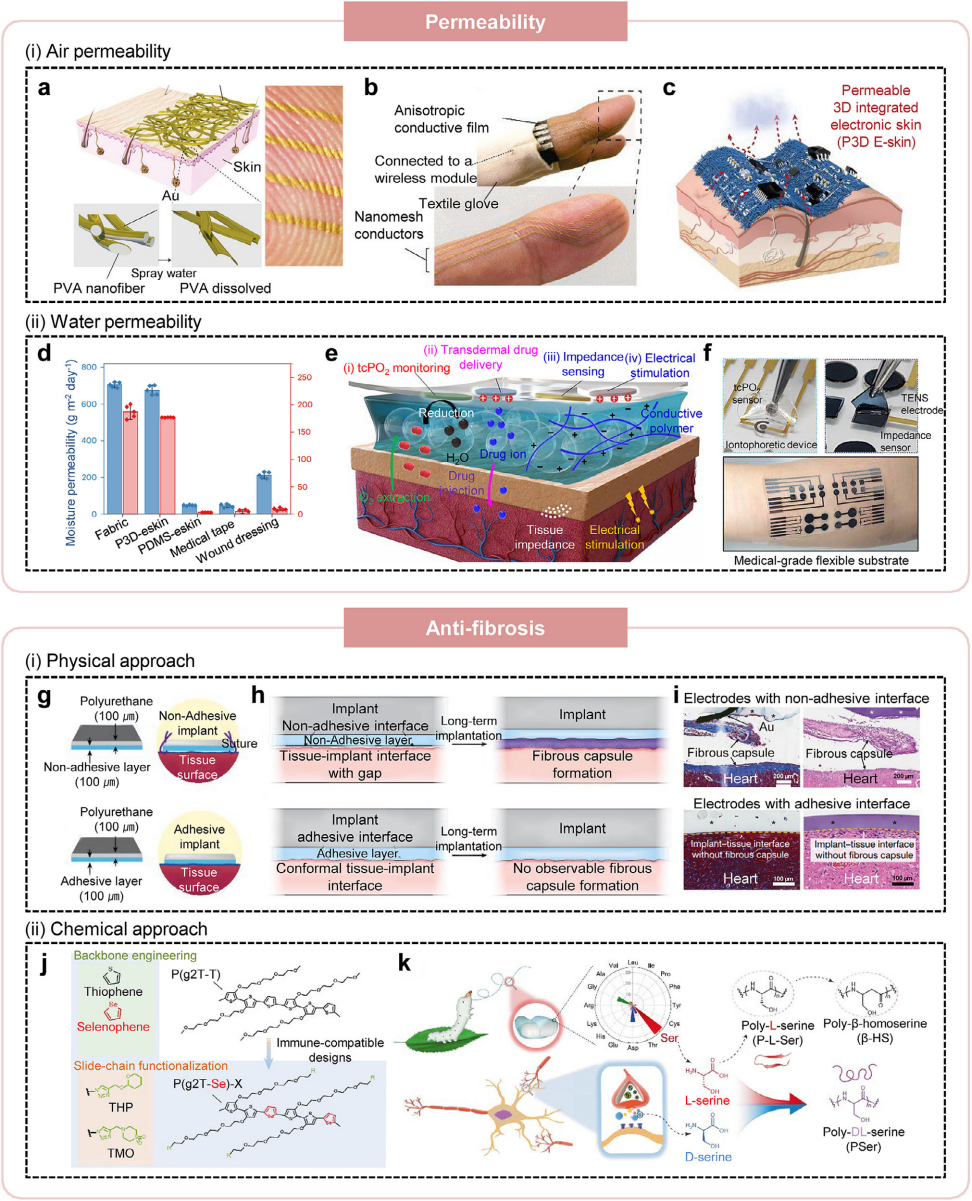
Biophysiologically adaptive bioelectronic interface with water permeability and anti‐fibrotic property. (a) Schematic illustration of the fabrication process for Au nanomesh conductors formed on electrospun PVA nanofibers and subsequently laminated onto skin surfaces. (b) Photograph of a nanomesh conductor conformally attached to a fingertip, demonstrating high skin adhesion and flexibility. Reproduced with permission [80]. Copyright 2017, Springer Nature. (c) Schematic representation of the air and moisture permeability of intrinsically permeable three‐dimensional electronic skin (P3D‐eskin). (d) Quantitative comparison of air and moisture permeability among wearable substrates, including P3D‐eskin, PDMS references, and commercial dressings. Reproduced with permission [87]. Copyright 2024, Springer Nature. (e) Schematic diagram of a hydrogel interfacial layer bridging human skin and wearable bioelectronics, enabling tcPO_2_ monitoring, impedance sensing, and transdermal drug delivery. (f) Photographs of hydrogel‐based wearable systems integrated on human skin for oxygen sensing and electrical stimulation. Reproduced with permission [84]. Copyright 2021, The American Association for the Advancement of Science. (g) Schematic illustration of a non‐adhesive implant consisting of a mock polyurethane device and a non‐adhesive layer. (h) Schematic illustration of an adhesive implant consisting of the mock polyurethane device and an adhesive interlayer that fills the tissue‐implant gap, thereby suppressing fibrotic encapsulation. (i) Representative histological images of non‐adhesive and adhesive implants on rat heart at day 28 post‐implantation, stained with Masson's trichrome (left) and hematoxylin and eosin (right). Asterisks indicate implants and yellow dashed lines mark implanttissue interfaces. Reproduced with permission [77]. Copyright 2024, Springer Nature. (j) Schematic representation of immune‐compatible semiconducting polymers engineered by substituting thiophene with selenophene to impart ROS‐scavenging capability and by introducing immunomodulatory THP and TMO side chains into the polymer backbone. Reproduced with permission [95]. Copyright 2025, Springer Nature. (k) Schematic illustration of stereochemically controlled poly‐DL‐serine (PSer) synthesized from L‐serine and D‐serine, designed as an anti‐fibrotic hydrogel inspired by natural serine enantiomer compositions in silk sericin and the human body. Reproduced with permission [93]. Copyright 2021, Springer Nature.

Flexible, wearable on‐skin electronics can conform to the curvilinear topography of human tissues, enabling stable physiological monitoring. However, long‐term use often induces skin irritation due to insufficient breathability. To address this issue, researchers from the Someya group developed nanofiber‐structured bioelectronics that simultaneously achieve mechanical conformality and high air permeability. (Figure 7a) [80]. Their PVA nanofiber conductors supported stable, long‐term temperature and pressure monitoring on the finger while minimizing inflammation risk (Figure 7b). Nonetheless, the simplicity of the nanofiber material system limited the fabrication of complex electrical components, constraining device integration density. To overcome these challenges, intrinsically permeable three‐dimensional electronics were developed by integrating liquid metal interconnects with elastomeric substrates (Figure 7c) [87]. Rigid chips were mounted onto stretchable liquid metal pathways embedded within an elastomeric matrix, forming a fabric‐like architecture with both high permeability of air and moisture, biocompatibility, and scalability. These permeable electronic skins further demonstrated multifunctionality, including sensing, electrical stimulation, and wireless communication.

Beyond air permeability, water permeability is essential for wearable electronics to maintain skin homeostasis and mitigate irritation. The porous architecture of intrinsically permeable electronic systems facilitates efficient moisture transport (Figure 7d), outperforming conventional biomedical interfaces in maintaining a balanced microenvironment. Recently, hydrogels have been incorporated as interfacial layers to further enhance water permeability and tissue compatibility [97]. Their hydrated polymer networks promote ionic and molecular transport at the biointerface, support high‐quality signal transduction, and reduce inflammatory responses by minimizing direct device‐skin contact. For example, Lim et al. developed a multimodal hydrogel system capable of oxygen concentration monitoring, impedance sensing, electrical stimulation, and transdermal drug delivery (Figure 7e) [84, 98]. In this platform, electrical communication is mediated through conductive hydrogel pathways, while electrochemical reactions associated with oxygen sensing and iontophoresis occur efficiently within the water‐rich environment, enabling integrated multimodal sensing and stimulation of physiological signals in vivo (Figure 7f).

#### 2.2.2 Anti‐Fibrosis

The introduction of foreign materials such as bioelectronic implants inevitably triggers activation of the host immune system, initiating a foreign body response. This immune cascade commonly culminates in the formation of dense fibrotic capsules that envelop the device, leading to pronounced degradation in signal quality, functional stability, and long‐term performance. Accordingly, mitigating this fibrotic encapsulation is essential for achieving chronic biocompatibility and maintaining reliable operation of implanted bioelectronic systems. Since anti‐fouling strategies are ultimately utilized for achieving the long‐term antifibrosis of the biomedical devices, we conceptually included the meaning of terminology “anti‐fouling” in this sub‐section.

One of the primary drivers of fibrotic encapsulation is the interfacial gap that forms between an implanted device and the surrounding tissues, as this void space facilitates fibroblast infiltration and subsequent collagen deposition. To address this challenge, physical strategies that promote conformal integration with curved tissue surfaces have been developed (Figure 7g). For instance, Wu et al. engineered an adhesive interlayer designed to eliminate the tissue‐implant gap, thereby effectively suppressing fibrotic capsule formation (Figure 7h) [77]. In multiple animal models, this approach significantly reduced inflammatory cell infiltration and collagen accumulation at the adhesive interfaces compared with non‐adhesive controls, demonstrating robust anti‐fibrotic performance (Figure 7i).

However, purely physical strategies may be insufficient for long‐term implantation, as natural organ motion and persistent mechanical stress can eventually cause device detachment from tissue surfaces [99]. To address this limitation, chemical approaches have been developed to suppress macrophage activity and downregulate inflammatory biomarkers. Li et al. incorporated selenophene units into the polymer backbone and cyclosulfone functionalities into the side chains, collectively attenuating inflammatory signaling (Figure 7j) [95]. The selenophene moieties endowed the material with reactive oxygen species‐scavenging capability, while the cyclosulfone groups further reduced pro‐inflammatory responses. In parallel, stereochemical modulation has emerged as a promising anti‐fibrotic design principle. Hydrogels synthesized from mixed‐enantiomer polyserine exhibited markedly reduced fibrotic encapsulation compared with single‐enantiomer formulations, resulting in lower cytokine expression and diminished inflammatory cell infiltration (Figure 7k) [93]. Together, these findings highlighted how chemical and stereochemical engineering can effectively mitigate fibrosis and promote the long‐term, stable integration of bioelectronic devices with soft tissues.

#### 2.2.3 Tissue Adhesion

As discussed above, establishing a seamless tissue‐device interface through adhesive materials is essential for preserving the long‐term performance of bioelectronic systems by preventing fibrotic encapsulation. Beyond their anti‐fibrotic benefits, tissue‐adhesive interfaces ensure stable device fixation at the target site, thereby enhancing signal reproducibility and improving the reliability of electrical and mechanical coupling between the device and surrounding tissues. In contrast, detachment arising from dynamic tissue motion can induce mechanical failure, increase interfacial impedance, and cause significant signal instability, ultimately compromising bioelectronic functionality.

**FIGURE 8:**
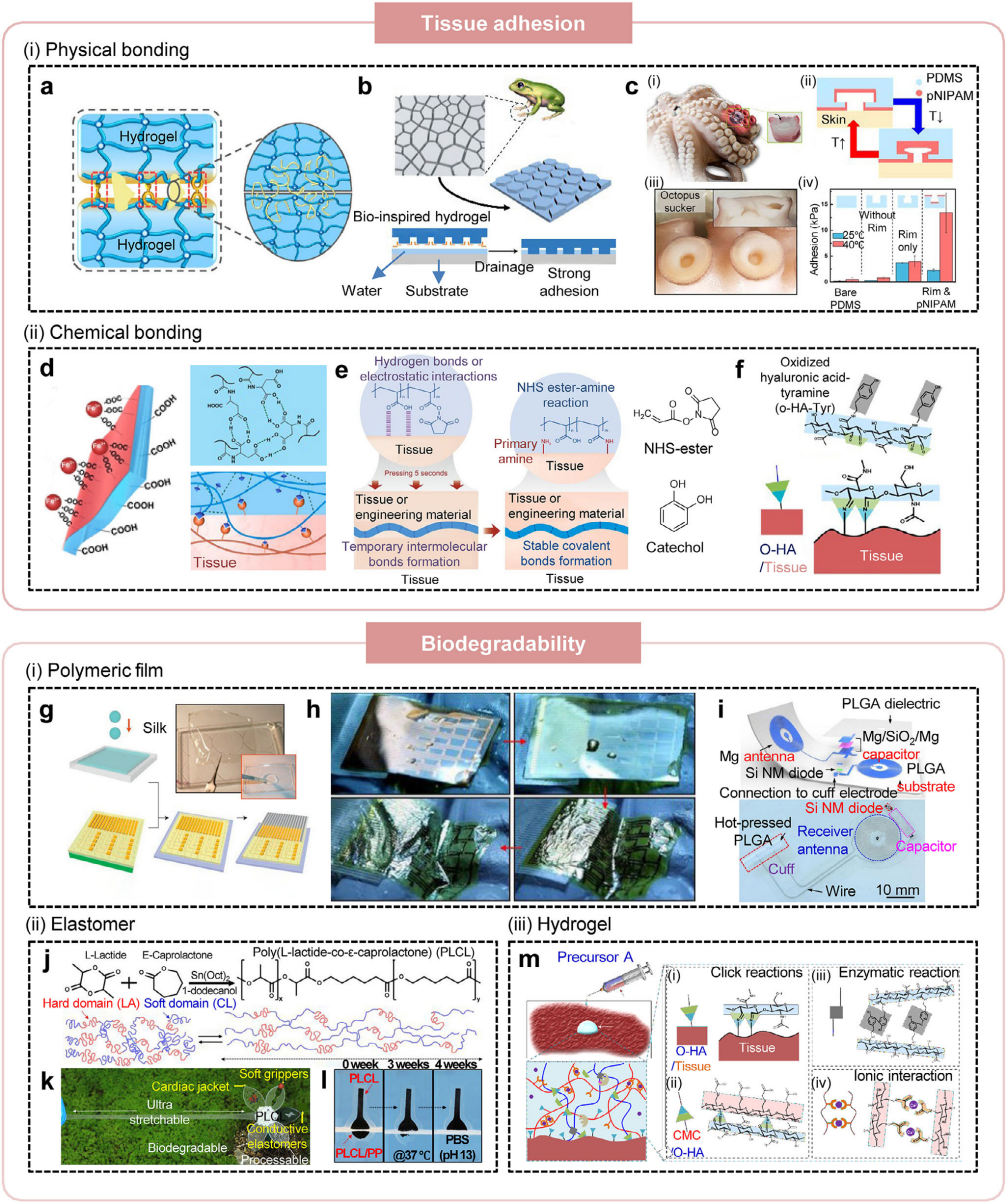
Biophysiologically adaptive bioelectronic interface with tissue adhesion and biodegradability. (a) Schematic illustration of polymer chain interpenetration between two hydrogel networks. Entangled polymer chains form a double‐network structure that resists separation without substantial external force, enabling physical bonding at the interface. Reproduced with permission [86]. Copyright 2023, American Chemical Society. (b) Structural characterization of tree‐frog toe pads used as inspiration for the design of biomimetic hydrogel patches with hierarchical microstructures that enhance interfacial contact and adhesion. Reproduced with permission [85]. Copyright 2021, American Chemical Society. (c) (i–iv), Bioinspired suction‐based adhesion mechanism of octopus tentacles. (i) Photograph of O. vulgaris tentacles with inset showing their anatomical cross‐section. Reproduced with permission [81]. Copyright 2017, Springer Nature. (ii) Temperature‐dependent reversible adhesion behavior. (iii) Optical image of octopus sucker with cross‐sectional view (inset). (iv) Structure‐dependent adhesion mechanism illustrated by schematic cross‐sections of artificial suction patches. Reproduced with permission [82]. Copyright 2018, American Chemical Society. (d) Schematic overview of PAASP hydrogel adhesion to tissues via hydrogen‐bonded crosslinked networks, and the corresponding Janus hydrogel patch exhibiting anisotropic adhesion. Reproduced with permission [90]. Copyright 2023, Elsevier. (e) Chemical tissue‐anchoring strategies. Formation of covalent amide bonds between NHS‐ester groups of double‐network hydrogel and primary amine groups on tissue surfaces, yielding thin, tough hydrogel layers with strong adhesion (left). Catechol and NHS‐ester functional groups are representative moieties that can form covalent bonds with tissues (right). Reproduced with permission [74]. Copyright 2019, Springer Nature. (f) Schematic illustration of in situ gelation and adhesion of o‐HA‐Tyr hydrogel on tissue surfaces via click‐chemistry‐mediated crosslinking. Reproduced with permission [75]. Copyright 2022, American Chemical Society. (g) Fabrication of degradable silk fibroin films by casting and drying on PDMS substrates to form thin, flexible polymeric layers for transient electronic systems. (h) Photographs showing enhanced conformal contact of silk‐based electrode arrays on a brain model as the overall device thickness decreases. Reproduced with permission [19]. Copyright 2010, Springer Nature. (i) Schematic illustration and photograph of a biodegradable nerve‐stimulation platform composed of PLGA substrate and magnesium‐based electronic components, enabling wireless power harvesting and natural degradation after therapy. Reproduced with permission [79]. Copyright 2018, Springer Nature. (j) Molecular structure of PLCL elastomer synthesized from biocompatible cyclic esters and its reversible chain motion during tensile stretching. (k) PLCL elastomer‐based soft device illustrating stretchability, degradability, and processability for bio‐integrated and soft‐robotic applications. Reproduced with permission [76]. Copyright 2023, Springer Nature. (l) Time‐lapse dissolution behavior of PLCL/PP‐based sensing probes under accelerated degradation conditions. (m) Schematic illustration of an injectable and biodegradable hydrogel exhibiting in situ gelation and tissue adhesion via multiple crosslinking mechanisms‐including click chemistry, enzymatic reactions, and ionic interactions. Reproduced with permission [75]. Copyright 2022, American Chemical Society.

Physical strategies for imparting adhesion to soft bioelectronic interfaces offer broad applicability across diverse polymer systems. The simplest approach relies on polymer chain entanglement, in which high‐molecular‐weight polymers interpenetrate with neighboring chains to form a double‐network structure that resists separation without substantial external force (Figure 8a) [86]. When such polymers interlock with biopolymers in tissues, the resulting interfacial network provides effective tissue adhesion. To achieve stronger and more reliable adhesion while maintaining material versatility, mechanical interlocking strategies inspired by biological architectures have been explored. Ho et al. introduced a biomimetic design replicating the hierarchical micro‐ and nanoscale structures found in the feet of frogs and geckos, where the expanded effective contact area enhances van der Waals interactions and promotes robust attachment. Using customized fabrication protocols, similar hierarchical topographies were reproduced in synthetic interfaces (Figure 8b) [85]. Related efforts have taken inspiration from octopus suckers, whose hollow cavities undergo volumetric deformation to generate negative pressure and reinforce adhesion [81]. This suction mechanism has been emulated using thermoresponsive polymers such as PNIPAM. (Figure 8c) where temperature‐induced volumetric transitions enable reversible suction‐based adhesion, providing strong yet detachable bonding at tissue interfaces (> 10 kPa) [82].

Although physical adhesion strategies can be broadly implemented across diverse hydrogel systems, their interfacial strength is inherently limited. To achieve more robust and durable coupling with biological tissues, chemical anchoring has been widely explored [100]. Hydrogen bonding, which is formed among amine, carboxylic acid, and hydroxyl groups, can be readily introduced by grafting these functional groups onto polymer backbones (Figure 8d) [90]. However, hydrogen bonding alone typically lacks the strength required for long‐term tissue adhesion. To address this limitation, in situ chemical crosslinking reactions have been employed.

Yuk et al. utilized EDCNHS chemistry to reinforce hydrogel‐tissue adhesion, wherein NHS‐ester‐functionalized hydrogels react with tissue amine groups to form stable amide linkages directly at the interface (Figure 8e) [74, 101]. Catechol grafting further enhances adhesion through synergistic hydrogen bonding and metal‐coordination interactions, enabling robust integration even under physiological conditions. Additional biorthogonal strategies have also been developed; for instance, hydrogels functionalized with aldehyde groups form imine linkages with primary amines on tissue biomolecules via rapid Schiff base reactions (Figure 8f) [75]. This spontaneous covalent coupling yields strong and stable tissue adhesion (∼ MPa), demonstrating high interfacial strength and long‐term durability.

#### 2.2.4 Biodegradability

Recent efforts have explored material design and engineering strategies that enhance long‐term biocompatibility for chronically implanted systems. Nonetheless, because both the device and its interfacing materials remain foreign entities, the potential for adverse biological responses persists as long as they remain in the body. To mitigate these risks, biodegradable interfaces and devices have been developed, allowing the system to safely resorb after completing its intended functional period [58, 59]. Representative examples span a broad range of biodegradable platforms, including polymers, elastomers, and hydrogels, each tailored to provide transient yet reliable operation before undergoing programmed degradation.

Degradable polymeric films have been widely investigated as sacrificial substrates for transient bioelectronics. A representative example is silk fibroin, a naturally derived and cytocompatible polymer that poses minimal toxicity concerns. Complex electronic circuits can be fabricated on silk films and subsequently transferred onto curvilinear tissue surfaces, enabling conformal device placement (Figure 8g) [19]. Following implantation, gradual enzymatic degradation of the silk substrate promotes seamless integration between the electronic components and the underlying tissue, with conformality further improving as the overall device thickness decreases (Figure 8h). However, silk‐based substrates offer limited tunability in degradation kinetics, which restricts their applicability for time‐specific therapeutic interventions. To attain more precise control over degradation behavior, synthetic copolymers such as poly(lactic‐co‐glycolic acid) (PLGA) have been employed. By adjusting the ratio of lactic to glycolic acid, the degradation period can be finely regulated. For example, a biodegradable nerve stimulation platform fabricated from hot‐pressed PLGA was demonstrated, wherein the substrate interfaced directly with neural tissue and gradually resorbed over the course of treatment (Figure 8i) [79].

Degradable polymers can also be engineered into elastomeric or hydrogel networks, and the combination of elasticity with controlled biodegradability significantly broadens the functional scope of soft bioelectronics. For example, a copolymeric elastomer based on poly(L‐lactide‐co‐ε‐caprolactone) (PLCL) was developed in which the lactide segments contribute rigidity while the caprolactone domains impart elasticity (Figure 8j) [76]. These complementary mechanical characteristics yield exceptional stretchability under external strain (Figure 8k), and accelerated in vitro studies verified the material's programmed biodegradability (Figure 8l). In parallel, Cha et al. reported a biodegradable hydrogel exhibiting injectability, adhesiveness, and stretchability. Through the design of a double‐network polymer and incorporation of functional moieties, in situ gelation was achieved via four concurrent reactions, enabling the formation of a multifunctional hydrogel interface suitable for transient biomedical applications (Figure 8m).

## 3 Multi‐Functional Adaptive Biointerfaces for Wearable and Implantable Applications

To accommodate the dynamic environments and continual structural changes of individual biological tissues, imparting multifunctionality to soft HMIs has become a central design priority, and diverse strategies are being actively explored [102, 103, 104]. For wearable soft HMIs, multifunctionality is particularly critical, as stable, high‐fidelity interfacing with moving and perspiring skin requires simultaneous acquisition of biopotentials, strain, temperature, and chemical signals, along with on‐body actuation capabilities such as haptic feedback, thermal therapy, and transdermal drug delivery. These functions must be coupled with reliable power management, wireless communication, and long‐term user comfort. Integrating these roles into a single, conformal platform reduces motion artifacts, eliminates rigid interconnects, and enables closed‐loop feedback during daily activities. This technological trajectory has been accelerated by advances such as shape‐programmable ultrathin laminates [105, 106]; materials with anti‐swelling, anti‐drying, and vapor‐permeable characteristics that preserve skin “breathing” [107, 108]; self‐healing conductive networks based on PEDOT:PSS, MXenes, or liquid‐metal microchannels that maintain low interfacial impedance [109, 110, 111]; tissue‐adhesive yet breathable encapsulants [112, 113]; and printed or stretchable radios [114], energy harvesters (triboelectric and thermoelectric) [115, 116], and near field communication (NFC)/bluetooth modules capable of on‐device analytics [117].

For implantable soft HMIs, achieving multifunctionality through the integration of multimodal sensing, electrical/chemical/mechanical stimulation, localized drug release, and secure telemetry is essential for closed‐loop therapy while maintaining a stable, minimally perturbative interface with surrounding tissues over extended periods. Anti‐fibrotic and biodegradable architectures, including zwitterionic or extracellular matrix‐mimetic surfaces, transient Mg/Si conductors, and silk‐, gelatin‐, or hyaluronic acid‐based matrices, help preserve low and stable tissue impedance while eliminating the need for explantation [77, 118, 119]. Injectable or deployable formats further minimize surgical trauma [120, 121], and chemically bonded hydrogel‐elastomer junctions safeguard the interface from delamination during organ motion [122, 123]. Complementary enabling technologies include bioresorbable energy storage [124] and ultrasound/NFC power delivery [125], microfluidic drug reservoirs integrated with electrical stimulation [126], photolithography‐compatible hydrogels that support patterned microelectrode fabrication [127], and scalable additive manufacturing approaches that co‐pattern soft sensors, actuators, and interconnects [128]. Collectively, these advances are paving the way for soft HMIs that not only conform and endure, but also sense, interpret, and actuate as unified bio‐integrated platforms across both wearable and implantable applications.

### 3.1 Wearable Bioelectronics With Multi‐Functional Adaptive Interface

**FIGURE 9:**
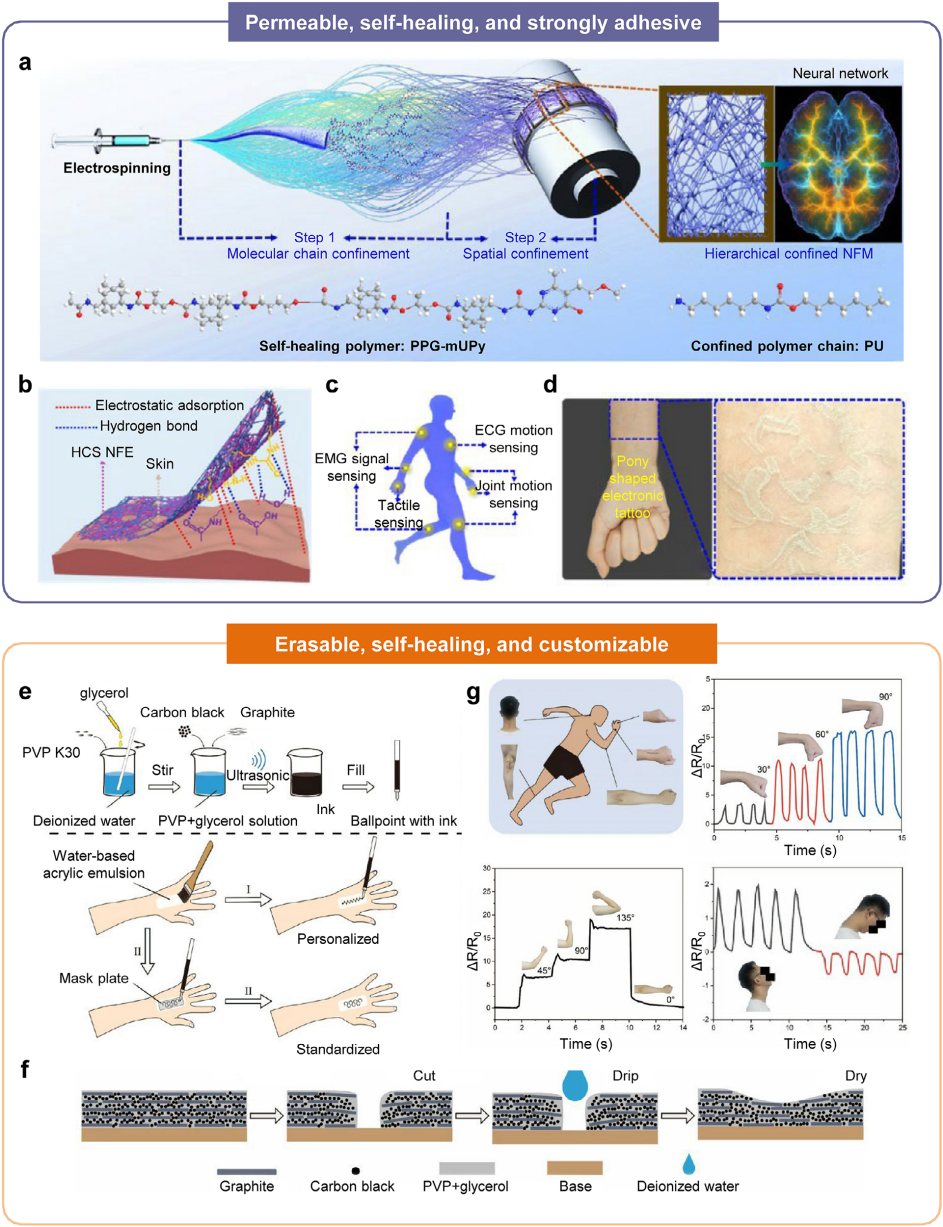
Wearable bioelectronics with multi‐functional adaptive interface. (a) Schematic illustration of the hierarchical confined nanofibrous membrane structure. (b) Adhesion mechanism of the membrane on skin. (c) Schematic illustration of electrophysiological signal monitoring using the skin‐like electrodes. (d) Photographs of the ultra‐thin, self‐healing skin‐like electrode attached to the arm. Reproduced with permission [129]. Copyright 2024, American Chemical Society. (e) Schematic illustration showing the fabrication and application processes of the erasable conductive ink. (f) Schematic explanation of the self‐healing process. (g) Biosignal recording capability of the penned device during daily activities. Reproduced with permission [132]. Copyright 2025, American Chemical Society.

Imparting multiple functionalities such as stretchability, permeability, and self‐healing within a single wearable HMI platform remains inherently challenging, largely due to the material trade‐offs that arise when engineering abiotic‐biotic interfaces. A representative dilemma is the difficulty of creating ultra‐thin, breathable electrodes that also possess intrinsic self‐healing capability; in nanofibrous systems, the strong inter‐fiber interactions required for mechanical robustness often destabilize supramolecular dynamics and suppress the autonomous bond exchange needed for healing. In a recent study, Zhu et al. addressed this challenge through a hierarchical confinement strategy that simultaneously modulates molecular‐scale and fiber‐scale interactions to stabilize dynamic hydrogen bonding within electrospun fibers (Figure 9a) [129]. This approach yielded an ultra‐thin, neural‐net‐like nanofibrous membrane that maintained supramolecular self‐healing while preserving the open porosity necessary for epidermal breathability. When integrated into a biomimetic epidermal electrode, the membrane exhibited excellent mechanical and electrical stability during long‐term on‐skin evaluation. The neural‐net architecture increased effective contact area on microscopically rough, mobile skin, enabling secure attachment with minimal interfacial energy and mitigating pressure‐induced irritation. Meanwhile, hierarchical confinement preserved a compliant, low‐modulus fiber surface capable of draping into shallow epidermal creases (Figure 9b), thereby sustaining intimate contact during dynamic motion. As a result, the electrodes enabled high‐fidelity real‐time electrophysiological monitoring with negligible burden to the underlying tissue (Figure 9c) and showed no adverse effects on skin health. These findings demonstrated that hierarchical confinement is a powerful design principle for reconciling self‐healing dynamics with nanofiber stability, enabling the creation of durable, imperceptible, and biocompatible epidermal interfaces (Figure 9d).

Sheng et al. developed a self‐healable hydrogel coating for porous substrates using a physically cross‐linked double‐network (DN) hydrogel synthesized via a plasma‐electrolysis Process [130]. The DN architecture generated a dense hydrogen‐bonding network that enabled high self‐repair efficiency while allowing the hydrogel to conformally infiltrate and coat the substrate's porous microstructure. In this approach, plasma electrolytic oxidation was first used to texture the substrate surface, followed by in situ DN‐hydrogel formation within the pores. The resulting soft HMI was systematically evaluated through electrochemical impedance spectroscopy and polarization tests in chloride media, alongside self‐healing assays that quantified recovery of mechanical and electrochemical performance. The coated interfaces exhibited markedly improved corrosion resistance compared to uncoated porous substrates and demonstrated rapid self‐healing, with efficiencies reaching ∼86% within 45 min, which was mainly attributed to robust supramolecular interactions within the DN network. These findings highlighted a practical and scalable strategy for forming conformal, damage‐tolerant hydrogel interfaces on porous architectures, enabling high‐performance soft HMIs with enhanced durability.

For long‐term use of wearable HMIs, achieving biocompatibility and antimicrobial protection is as critical as ensuring intrinsic softness, breathability, and self‐healing capability. Xu et al. reported a permeable and antimicrobial HMI platform based on liquid‐metal (LM) conductors, enabled by a phase‐separation synthesis that produced porous LM‐elastomer composites [131]. In this architecture, a microcellular elastomer network mechanically confined LM droplets, suppressing flow and leakage while lowering the electrical percolation threshold, thereby reducing LM consumption without compromising conductivity. Simultaneously, ε‐poly‐L‐lysine was incorporated into the matrix to impart broad‐spectrum antimicrobial activity. The resulting composites exhibited tissue‐like compliance, large stretchability, deformation‐stable conductivity, high vapor permeability, and magnetic resonance imaging compatibility, and were integrated into skin‐interfaced bioelectronics capable of continuous cardiac electrical and mechanical monitoring as well as electrical stimulation during motion. This study demonstrated a manufacturable materials strategy that mitigates LM leakage and introduces intrinsic antimicrobial function while maintaining mechanical and electrical robustness, advancing LM composites toward safe, durable, and hygienic epidermal bioelectronic interfaces.

More recently, Zhu et al. introduced a direct‐writing strategy for erasable, reconfigurable, and multifunctional on‐skin HMIs, addressing three persistent limitations of current epidermal systems: (1) reliance on complex prefabrication and transfer printing, (2) limited customizability due to fixed single‐function layouts, and (3) poor reusability once worn [132]. The authors engineered a skin‐adhered, flexible coating derived from a conductive ink that served as a write‐once and readily erasable substrate. This hydrophilic composite ink, prepared from graphite, carbon black, polyvinylpyrrolidone (PVP K30), glycerol, and water, exploited PVP as a multifunctional component: a dispersant for carbon fillers, a non‐ionic surfactant lowering surface tension for uniform deposition, and a bonding agent that immobilizes fillers upon drying. Glycerol further tuned viscosity, stabilized dispersion, and enhanced adhesion and deformation resistance after solvent evaporation. Using a ballpoint‐pen‐style conductor, users could freehand‐draw circuit traces and sensor architectures directly onto the coated skin and subsequently erase or reconfigure them as needed (Figure 9e). The system's self‐healing ability emerged from water‐mediated rebridging of the graphite/carbon‐black percolation network within the PVP/glycerol matrix on a water‐based acrylic emulsion substrate (Figure 9f). The resulting penned electronics were ultrathin, breathable, and conformal, maintained stable signal quality during daily activities (Figure 9g), and could be repeatedly erased and rewritten to alter sensor topology or repair damage. In all, this work demonstrated a practical pathway for fabricating customizable, multifunctional, and reusable on‐skin HMIs directly at the point of care without relying on conventional transfer processes or adhesives.

### 3.2 Implantable Bioelectronics With Multi‐Functional Adaptive Interface

**FIGURE 10:**
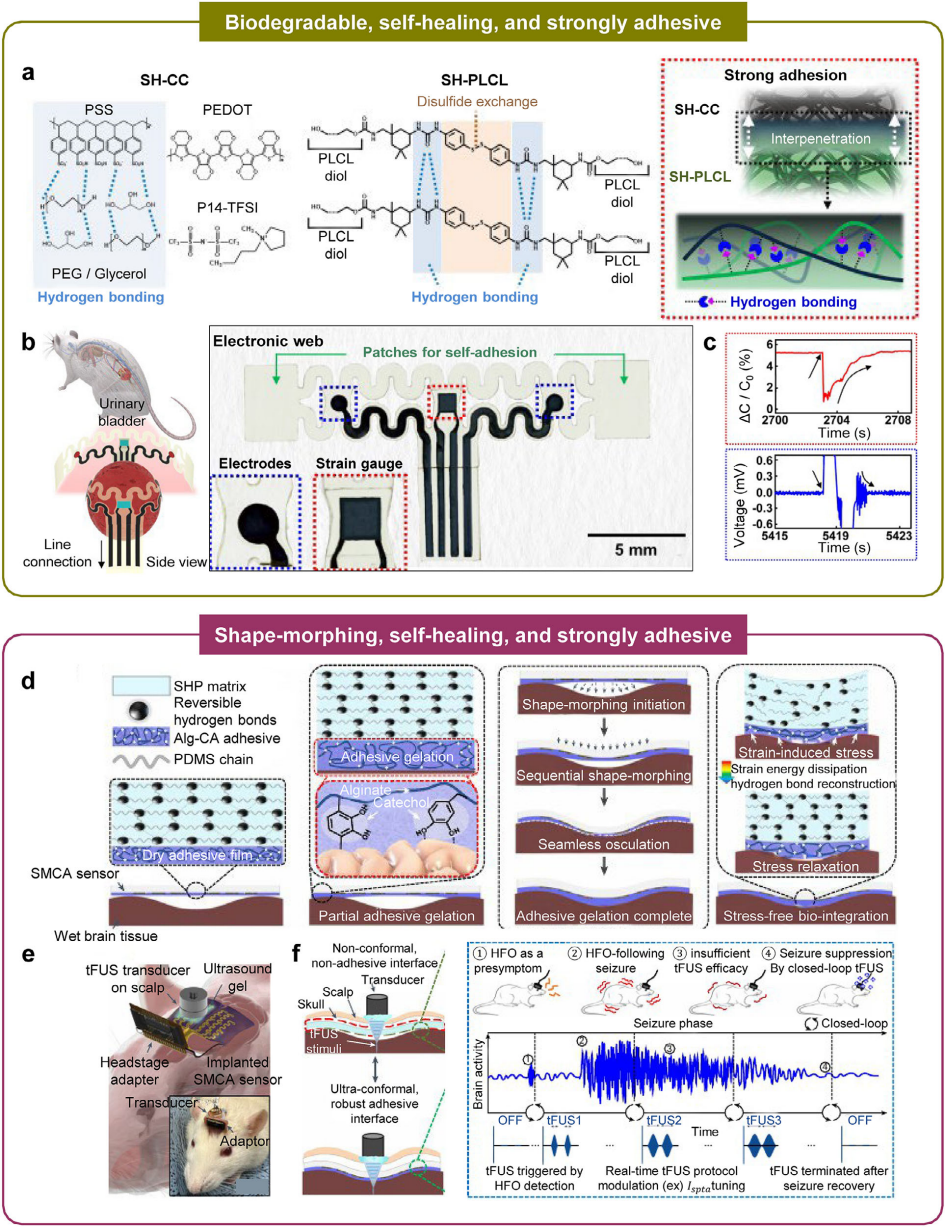
Implantable bioelectronics with multi‐functional adaptive interface. (a) Schematic illustration of the chemical structures of SH‐CC and SH‐PLCL containing hydrogen bonding for self‐healing capability. (b) Schematic and photographic demonstration showing the integration of electronic web device on urinary bladder of the mouse. (c) Electrical recovery of the electronic web after scratching with a razor blade. (d) Schematic illustration showing the shape‐morphing mechanism of the implantable device. Reproduced with permission [133]. Copyright 2024, American Association for the Advancement of Science. (e) Description of the actual implantation system for closed‐loop neural recording and feedback stimulation. (f) Schematic illustration showing the successful closed‐loop operation of pre‐seizure detection and ultrasound dose for seizure suppression in an awake rodent epilepsy model. Reproduced with permission [135]. Copyright 2024, Springer Nature.

Multi‐functionalities such as biocompatibility, biodegradability, self‐healing, modulus matching with target biological tissues, and strong adhesion are important for implantable HMIs, thus posing even more difficulties in terms of materials selection and device design/fabrication. Jang et al. engineered a biodegradable, stretchable, and intrinsically self‐healing conductor to realize multifunctional implantable HMI capable of sensing and therapy in a single platform [133]. In specific, the authors developed a two‐layer conductor comprising a self‐healing elastomer (SH‐PLCL) featuring dynamic disulfide exchange and H‐bonding, and a self‐healing conductive composite (SH‐CC) based on PEDOT:PSS plasticized with P14[TFSI] (stretchability enhancer) and polyethylene glycol/glycerol (self‐healing and conductivity enhancers) (Figure 10a). A strong adhesion between the two layers was achieved via interpenetration and secondary bonding across the SH‐PLCL/SH‐CC interface. The authors demonstrated a system‐level in vivo evaluation of their implantable device, equipped with strain sensing, electromyography recording, and electrical stimulation modules in a single platform, on the urinary bladder of a mouse (Figure 10b). The in vivo test revealed a rapid, room‐temperature recovery (∼2 min) of electrical function after full cuts, high conductivity (∼10^3^ Scm^−^^1^) and stretchability (∼500%) sustained through repeated damage, strong interlayer adhesion from polymer interpenetration, aqueous biodegradation behavior of SH‐PLCL, and a self‐healing implant that improved bladder voiding efficiency while maintaining stable signals during repetitive organ motion (Figure 10c). This study validated a materials architecture that unified biodegradability, mechanical resilience, and closed‐loop diagnostic/therapeutic operation for multi‐functional implantable devices.

Jung et al. reported a biocompatible and intrinsically stretchable organic field‐effect transistor (sOFET) platform aimed at multi‐functional implantable interface capable of executing basic on‐device logic while enduring physiological strains and biofluids [134]. The targeted multi‐functionality was the co‐integration of mechanically compliant, low‐voltage OFETs with stretchable, corrosion‐resistant electrodes into active‐matrix and logic circuits (inverter, NOR, NAND) that operate in vivo, thereby moving beyond passive sensing toward local signal processing inside tissue. To accomplish this goal, the authors blended a high‐mobility semiconducting polymer (DPPT‐TT) with a medical‐grade bromobutyl rubber (BIIR) and vulcanized the elastomer (sulfur/DPTT/stearic acid) to form a nanofiber‐percolated semiconductor within an elastic matrix. This enabled crack‐free operation to 100% strain and stable mobility over 1000 cycles, while Ag/Au dual‐layer metallization provided stretchable contacts with sweat‐resistant, biofluid‐stable performance (72 h in artificial sweat). Active‐matrix arrays and pseudo‐CMOS logic were fabricated to validate their performance under strain and in aqueous media, followed by a structured biocompatibility program including in vitro assays with human dermal fibroblasts and macrophages, antibacterial tests, and a 30‐day subcutaneous mouse study that analyzed inflammatory markers and fibrous capsule thickness. In addition, the sOFETs and circuits retained the mobility and on/off ratio up to 50% strain and after 10 000 cycles, and remained stable after soaking in deionized water, saline, and PBS. Subcutaneously implanted logic circuits maintained switching characteristics over several days, evidencing operational viability of elastomeric OFET logic inside living tissue. Thus, this work established a materials blueprint that enabled stretchable OFET logic with validated in vitro/in vivo biocompatibility, addressing long‐standing weaknesses of ion‐gated OECTs (high off‐currents/crosstalk) for implantable computation.

In another work by Lee et al., a unique shape‐morphing HMI that could strongly adhere to a curved, wet cortex was reported, capable of closed‐loop transcranial focused ultrasound (tFUS) neurostimulation [135]. In their work, a catechol‐conjugated alginate hydrogel was used to provide instantaneous, robust cortex adhesion, and a viscoplastic, self‐healing polymer substrate was developed to endow shape‐morphing conformity and mechanical durability (Figure 10d). A stretchable 16‐channel ECoG array was fabricated to demonstrate high‐fidelity sensing, where the sensors were coupled to a pulse‐controlled tFUS module for closed‐loop operation (Figure 10e). As a demonstration, awake rodent epilepsy models in which the system detected pre‐seizure high‐frequency oscillations (HFOs) and automatically adjusted ultrasound dose for seizure suppression were developed (Figure 10f). The results successfully showed that the cortex‐adhesive, shape‐morphing sensor maintained conformal, artifact‐resistant neural recordings during tFUS, reliably detected pre‐seizure HFOs in real time, and enabled closed‐loop seizure control with adaptive ultrasound dosing in awake animals, demonstrating a multi‐functional, materials‐engineered route to practical closed‐loop ultrasound neuromodulation.

## 4 Conclusion

In this review, we highlighted recent advances in adaptive HMI technologies for third‐generation bioelectronics, classifying them into mechano‐adaptive and biophysiologically adaptive modalities. We first described mechano‐adaptive strategies, which include shape‐programmable architectures, injectable systems, anti‐swelling designs, and intrinsic self‐healing mechanisms, and then outlined biophysiologically adaptive approaches such as controlled‐permeability interfaces, anti‐fibrotic chemistries, tissue‐adhesive layers, and biodegradable components. Representative wearable and implantable systems that integrate these adaptive interfaces were examined to illustrate their translational potential. Finally, we discussed remaining challenges and future research directions required to achieve stable, long‐term, and clinically viable bioelectronic platforms.

While current soft HMI systems exhibit remarkable progress, including mechano‐adaptive architectures and biophysiologically engineered surfaces, future interfaces must support far more complex, high‐dimensional functions that could be classified as technological innovation and clinical translation. In terms of clinical translation, in vivo stability and clinical reliability of the adaptive HMI remain a critical challenge. For example, degradation pathways under chronic physiological conditions, including hydrolytic, enzymatic, and oxidative processes, may affect the performance and functionality over operation periods. In addition to the progressive signal attenuation, the relevance of HMI with biosafety issues (e.g., chronic immune responses and foreign body reactions) should be presented. Future research should therefore proceed with various standardized reliability assessments from in vitro to in vivo experiments, including accelerated aging protocols, long‐term implantation studies, biofouling resistance testing, and mechanical‐electrical stability evaluation under cyclic physiological loading. Establishing unified validation frameworks will be essential to enable objective comparison across different types of HMIs.

The translational and regulatory considerations that govern clinical adoption are also required for commercialization. Sterilization compatibility (e.g., ethylene oxide, gamma irradiation), scalable and reproducible manufacturing strategies, device packaging and powering solutions, and compliance with established biocompatibility standards such as the ISO 10993 series must be considered with the early device design. Addressing these reliability, standardization, and regulatory dimensions will ultimately determine whether adaptive HMI can transition from promising prototypes to robust and clinically deployable technologies.

Realizing the technical innovation will require deeply multidisciplinary and convergent research. Integrating AI‐driven signal interpretation, autonomous closed‐loop control, and tissue‐specific biomimicry represents a particularly promising direction for creating interfaces that match the complexity and dynamic behaviors of living systems. Such convergence could enable bioresorbable platforms with programmable lifetimes, multimodal sensing‐actuation hybrids, and immune‐modulating interfaces that actively harmonize with host physiology. These future developments will allow next‐generation soft HMIs to coexist seamlessly with biological tissues, advancing personalized healthcare, real‐time diagnostics, and adaptive therapeutics, establishing a foundational blueprint for the future of human‐machine integration.

## Conflicts of Interest

The authors declare no conflicts of interest.

## Data Availability

The authors have nothing to report.
